# 3D Printing of Extracellular Matrix‐Based Multicomponent, All‐Natural, Highly Elastic, and Functional Materials toward Vascular Tissue Engineering

**DOI:** 10.1002/adhm.202203044

**Published:** 2023-04-25

**Authors:** Melis Isik, Ece Karakaya, Tugba Sezgin Arslan, Deniz Atila, Yasar Kemal Erdogan, Yavuz Emre Arslan, Hakan Eskizengin, Cemil Can Eylem, Emirhan Nemutlu, Batur Ercan, Matteo D'Este, Babatunde O. Okesola, Burak Derkus

**Affiliations:** ^1^ Stem Cell Research Lab Department of Chemistry Faculty of Science Ankara University Ankara 06560 Turkey; ^2^ Department of Engineering Sciences Middle East Technical University Ankara 06800 Turkey; ^3^ International Centre for Research on Innovative Bio‐based Materials (ICRI‐BioM) Lodz University of Technology Lodz 90924 Poland; ^4^ Biomedical Engineering Program Middle East Technical University Ankara 06800 Turkey; ^5^ Department of Biomedical Engineering Isparta University of Applied Science Isparta 32260 Turkey; ^6^ Regenerative Biomaterials Laboratory, Department of Bioengineering Faculty of Engineering Canakkale Onsekiz Mart University Canakkale 17100 Turkey; ^7^ Department of Biology Faculty of Science Ankara University Ankara 06560 Turkey; ^8^ Analytical Chemistry Division Faculty of Pharmacy Hacettepe University Ankara 06230 Turkey; ^9^ Bioanalytic and Omics Laboratory Faculty of Pharmacy Hacettepe University Ankara 06100 Turkey; ^10^ Department of Metallurgical and Materials Engineering Middle East Technical University Ankara 06800 Turkey; ^11^ AO Research Institute Davos Clavadelerstrasse 8 Davos Platz 7270 Switzerland; ^12^ School of Life Sciences, Faculty of Medicine and Health Sciences University of Nottingham University Park Nottingham NG7 2UH UK

**Keywords:** 3D printing, aorta grafts, decellularization, multicomponent hydrogels, vascular tissue engineering

## Abstract

3D printing offers an exciting opportunity to fabricate biological constructs with specific geometries, clinically relevant sizes, and functions for biomedical applications. However, successful application of 3D printing is limited by the narrow range of printable and bio‐instructive materials. Multicomponent hydrogel bioinks present unique opportunities to create bio‐instructive materials able to display high structural fidelity and fulfill the mechanical and functional requirements for in situ tissue engineering. Herein, 3D printable and perfusable multicomponent hydrogel constructs with high elasticity, self‐recovery properties, excellent hydrodynamic performance, and improved bioactivity are reported. The materials' design strategy integrates fast gelation kinetics of sodium alginate (Alg), in situ crosslinking of tyramine‐modified hyaluronic acid (HAT), and temperature‐dependent self‐assembly and biological functions of decellularized aorta (dAECM). Using extrusion‐based printing approach, the capability to print the multicomponent hydrogel bioinks with high precision into a well‐defined vascular constructs able to withstand flow and repetitive cyclic compressive loading, is demonstrated. Both in vitro and pre‐clinical models are used to show the pro‐angiogenic and anti‐inflammatory properties of the multicomponent vascular constructs. This study presents a strategy to create new bioink whose functional properties are greater than the sum of their components and with potential applications in vascular tissue engineering and regenerative medicine.

## Graphical abstract

3D‐printed, multicomponent, hyper‐elastic graft for vascular tissue engineering is developed. The bioink consists of decellularized aorta, tyramine‐modified hyaluronic acid, and alginate. The emerged graft induces angiogenesis, does not lead immunological reactions, and is perfusable.

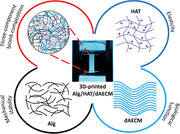

## 1 Introduction

There is an increasing need to reconstruct or replace damaged tissues due to disease, trauma, or surgery.^[^ ^1^, ^2^ ^]^ 3D printing is an emerging innovative technology for the creation of complex biological structures such as those that can be used as tissue analogues, implants, grafts, and regenerative biomaterials.^[^ ^3^, ^4^ ^]^ This technique presents a unique opportunity to reconstruct human tissues and organs with precise spatial control in a manner that mimics both the structures and functionalities of native tissues. To effectively biofabricate biomimetic constructs, printing materials also known as bioinks, including natural biopolymers (e.g., alginate^[^ ^5^ ^]^ and collagen^[^ ^6^ ^]^), functionalized natural biopolymers (e.g., tyramine‐modified hyaluronic acid^[^ ^7^ ^]^, methacrylated gelatin,^[^ ^8^ ^]^ and hyaluronic acid^[^ ^9^ ^]^) as well as synthetic polymers (e.g., methacrylated polyethylene glycol, polycaprolactone, and poly(lactic acid))^[^ ^10^, ^11^ ^]^ have been widely exploited. However, 3D printing is currently limited by the scarcity of bioinks with excellent printability, biocompatibility, and desired mechanical and bioactive properties for functional tissue constructs. 3D‐printed vascular constructs in particular require bioinks that are non‐toxic, non‐immunogenic, low cost, and able to support the flow of blood and withstand the pressures exerted by this flow.^[^ ^12^ ^]^ In addition, the native extracellular matrices (ECMs) of vascular tissues are a complex combination of multiple biomolecules each of which shows specific biological effects on the cellular behaviors.^[^ ^13^ ^]^ Considering the design requirements, including high tensile strength and viscoelasticity, fatigue resistance, biocompatibility, and suturability, amongst others, for an artificial vascular construct,^[^ ^14^ ^]^ many of the existing 3D‐printed architectures composed of single‐component materials cannot replicate the essential functions and structural features of the native vascular tissues.

Multicomponent 3D printing technique presents a unique opportunity to harness desired mechanical property and complex biological functions in 3D printing of high‐performance vascular tissue constructs.^[^ ^15^, ^16^ ^]^ Multicomponent hydrogels composed of naturally derived building blocks are attractive candidates for 3D printing due to their inherent bioactivity, high similarity to the native ECM, and excellent biocompatibility and biodegradability.^[^ ^17^, ^18^ ^]^ Alginate is a particularly important class of bioink for 3D printing technology owing to its inherent biocompatibility, biodegradability, and simple gelation by divalent ions.^[^ ^15^ ^]^ However, alginates have poor mechanical properties, lack functional epitopes for cell adhesion, and also lack long‐term stability which is essential for post‐printing shape fidelity.

To this end, there has been a growing interest in multicomponent strategies integrating the inherent properties of alginate with those of other natural biopolymers including hyaluronic acid, silk, collagen, gelatin, and chondroitin sulfate to create hydrogel materials with improved rheological characteristics and cell viability for extrusion‐based printing of tissue engineering constructs.^[^ ^19^, ^20^, ^21^ ^]^ Ouyang et al. (2015) developed a multicomponent system comprising methacrylated gelatin (GelMA), alginate, and fibrinogen.^[^ ^22^ ^]^ Combining radical polymerization of GelMA, multiple hydrogen bond interactions of fibrinogen, and ionic crosslinking of alginate, the printed multicomponent grid constructs displayed long‐term stability and high biocompatibility and are suitable for mammalian cell spheroids growth. To improve cell adhesion and bioprintability of low viscosity alginate‐based multicomponent hydrogels, Ning et al. integrated RGD modified alginate, hyaluronic acid, and fibrin through ionic crosslinking, enzymatic transformation, and hydrogen bond interactions.^[^ ^23^ ^]^ By fine‐tuning the printing parameters (temperature, printing pressure, and printing head speed) and crosslinking procedures including calcium ions and thrombin concentrations, the authors bioprinted Schwann cell‐laden layered grid constructs with high scaffold stability and distinguishable pores. This cell‐laden multicomponent bioink supported neurite outgrowth, suggesting a great potential for nerve tissue repair. Zhuo et al. integrated alginate, GelMA, and gelatin using both photopolymerization and ionic crosslinking to create multicomponent bioink for artificial blood vessels with mechanical and biological properties that matched the native tissues.^[^ ^24^ ^]^ The multicomponent vascular constructs showed excellent hemolysis, cell viability, and proliferation in vitro and promoted neo‐vascularization in vivo, suggesting their potential applications in vascular tissue engineering.

To design functional engineered constructs that can promote not just cell adhesion and proliferation but also display spatial orientation of native tissues, multiple bio‐instructive cues are required to be presented in space and time to support multiple biological processes mediating functional tissue growth.^[^ ^25^ ^]^ The use of tissue‐specific decellularized ECM (dECM) derived from native tissue is an emerging strategy to incorporate multiple biological functions into multicomponent 3D‐printed constructs.^[^ ^18^ ^]^ Gao et al. combined decellularized vascular‐tissue‐derived extracellular matrix with alginate to compensate the drawback of alginate to both enable the direct tube bioprinting and improve cell functions.^[^ ^26^ ^]^ The authors showed that endothelial progenitor cells and proangiogenic drugs (atorvastatin)‐loaded multicomponent construct exhibit enhanced survival and differentiation of endothelial cells, increased rate of neovascularization, and remarkably restored ischemic tissues when implanted into a nude mouse. Notwithstanding, multicomponent constructs generated with dECMs are far from meeting the mechanical property requirements for an ideal vascular construct; and hence, their usage is currently limited to simple grid constructs and fragile tubular materials.^[^ ^27^, ^28^, ^29^, ^30^ ^]^

Herein, we report the development of a new “breed” of multicomponent hydrogel bioinks to create elastic, perfusable, self‐recoverable, biocompatible, and bio‐instructive 3D‐printed constructs with molecular precision and spatial control. We demonstrate that this multicomponent bioink system, composed of alginate, hyaluronic acid–tyramine, and decellularized bovine aorta ECM (dAECM) can be used to print grids and tube‐like structures at clinically relevant length scales for tissue engineering without the need for an external support. By tuning the molecular composition and the gelation trigger factors, we can create a library of 3D printed constructs. We show that the multicomponent bioinks and the printed constructs containing dAECM enhance the survival and proliferation of primary human umbilical cord vein endothelial cells (HUVECs), suppress unfavorable foreign body response (anti‐inflammatory response), and stimulate vascularization of the implant in ovo using chorioallantoic membrane (CAM) assay. As a proof‐of‐concept, we show that our multicomponent 3D‐printed constructs can be used as a fluidic device, mimicking vascular tissue analogues, and can also be adapted for a broad range of tissue engineering applications.

## 2 Results and Discussion

### 2.1 Rationale for Materials Design

**Figure 1:**
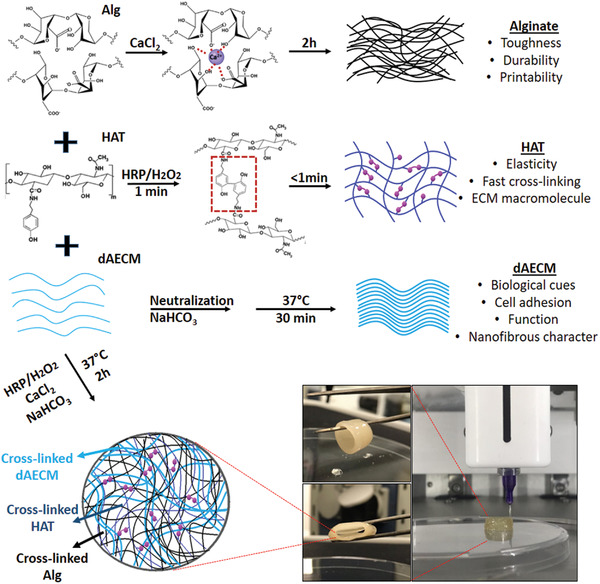
Hydrogel bioink design strategy. Schematic representation of molecular components and design strategies for multicomponent hydrogel bioinks as well as hydrogels of individual component. Each component brings its unique functional properties.

Advanced functional biomaterials with desired mechanical strength, elasticity, and bioactivity are potential materials for tissue regeneration and organ or tissue transplantation. We develop multicomponent bioink integrating the fast gelation kinetic of sodium alginate (Alg) by divalent ions, in situ crosslinking of tyramine‐modified hyaluronic acid (HAT),^[^ ^31^, ^32^ ^]^ and multiple biological functions and temperature‐dependent self‐assembly of dAECM. We reason that calcium (Ca^2+^) ions crosslinking of Alg through the egg‐box model presents opportunity for fast gelation during 3D printing.^[^ ^33^ ^]^ Similarly, we anticipate that excess Ca^2+^ ions will facilitate intermolecular interactions among Alg, HAT, and dAECM in the multicomponent bioink (**Figure** **1**). Horseradish peroxidase (HRP)/hydrogen peroxide (H_2_O_2_)‐mediated oxidative coupling of HAT and tyrosine residues on the dAECM will enable dityrosine bridge formation, which will further impart the multicomponent hydrogels with toughness and ability to withstand cyclic compressive loading, making them ideal bioinks for 3D printed constructs for blood vessels, skin, and muscles.^[^ ^34^, ^35^, ^36^, ^37^ ^]^ These orthogonal interactions endow the multicomponent bioinks with unparallel mechanical performance compared to the individual components. Incorporation of dAECM into the multicomponent system installs multiple bio‐instructive functions, which cannot be obtained from Alg and HAT individually or combined.

### 2.2 Decellularization of Aorta, Gelation of dAECM, and Cytocompatibility of dAECM Hydrogels

**Figure 2:**
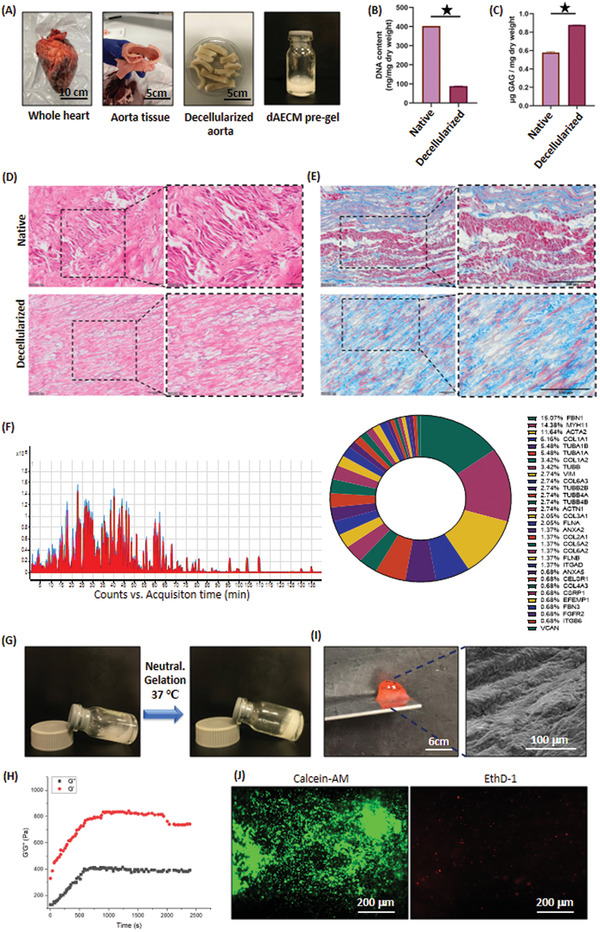
Decellularization and hydrogelation of aorta tissue. A) Macroscopic images showing the decellularization process. B) Residual dsDNA and C) sGAG content in dAECM in comparison with native aorta tissue (*p < 0.05). D) Histochemical staining of native aorta and dAECM with H&E indicating the removal of cytoplasm (cells) and nuclear materials. E) Histochemical staining of native aorta and dAECM with Masson's trichrome indicating complete removal of SMCs (stained red) and retention of collagen structure (stained blue). F) Mass spectrum and pie chart related to the proteomics analysis of dAECM and the relative percent of key ECM proteins. G) Macroscopic images of dAECM solution and sol‐to‐hydrogel transition upon neutralization and thermal induction. H) Time sweep rheology demonstrates the sol‐to‐gel transition of dAECM hydrogel (1% wt). I) SEM image showing the nano–micro fibrous architecture of dAECM (1% wt) hydrogel. J) Calcein‐AM/EthD‐1 double staining fluorescent images showing the live (green) and dead (red) cells seeded on the dAECM hydrogel.

We used sodium dodecyl sulfate (SDS) and dimethyl sulfoxide (DMSO) to prepare dAECM from native aorta samples obtained from bovine as previously reported elsewhere.^[^ ^38^ ^]^ To assess the quality and purity of dAECM (**Figure** **2**A), first we measured the double‐stranded DNA (dsDNA) content of dAECM to ensure significant removal of genetic materials. The DNA content of native aorta tissue was 400 ng mg^−1^ dry tissue. The DNA content was significantly reduced by 75% (100 ng mg^−1^ DNA) after decellularization (Figure 2B). We used quantitative and qualitative techniques to determine sulfated glycosaminoglycans (sGAGs) content of dAECM to ascertain structural stability. The sGAG content after decellularization process increased by 25% (≈0.8 *µ*g sGAG/mg dry weight) compared to the native tissue (≈0.6 *µ*g sGAG/mg dry weight), suggesting that a significant amount of sGAG was retained in dAECM with little or no cellular and nuclear materials remaining (Figure 2C). Higher level of sGAG after decellularization process can be attributed to the relative weight that takes into account the dry weight of the decellularized ECM. Histological examination with hematoxylin and eosin (H&E) confirms complete elimination of cytoplasmic components, intact cells or cell remnants, and nuclei in dAECM after decellularization compared to the native aorta (Figure 2D). Moreover, Masson's trichrome staining showed that smooth muscle cells (SMCs, stained in red) and collagen (stained in blue) are the predominant constituents of the native aorta. The decellularized aorta is void of SMCs, but the collagen fibers were preserved with no obvious structural degradation (Figure 2E). We used proteomics to identify the protein contents of our dAECM (Figure 2F). Our data revealed the abundance of functional ECM proteins and growth factors (Table S1, Supporting Information). The presence of annexin, cadherin, vimentin, a broad range of collagen family, integrin family proteins, and tubulin family proteins (relative percent of identifier peptides and peptide‐specific mass spectra can be found in Figure S1, Supporting Information) shows that dAECM is a potential bio‐instructive material (Figure 2F, pie chart). More importantly, the presence of blood vessel‐specific proteins, such as aortic smooth muscle actin, coagulation factor V, destrin, fibrillin family proteins, and myosin‐11, indicates that dAECM is a potential building block to fabricate vascular tissue analogues.

To demonstrate the gelling ability of an aqueous solution of dAECM (1% wt), we used temperature switching (∆*T*) mechanism following the initial neutralization with sodium bicarbonate. This gelation protocol generated self‐supporting hydrogels (Figure 2G). It is noteworthy that an attempt to create dAECM hydrogels without the neutralization step proves abortive. We used time sweep oscillatory rheology to show the kinetic of gelation of dAECM upon gradual cooling after the initial heating up to 37 °C. Both the storage (*G*′) and loss (*G*″) moduli increased with time until a plateau was attained. The *G*′ value (700 Pa) of dAECM hydrogels was greater than the *G*″ value (380 Pa) (Figure 2H), indicating that dAECM can form an ideal hydrogel, albeit with poor mechanical property. The hydrogels formed nanofibrillar network as revealed by scanning electron microscopy (SEM) (Figure 2I), suggesting that the molecular component of dAECM still retained its propensity to hierarchically self‐assemble into macroscopic hydrogels in a kinetic and thermodynamic manner. To assess the suitability of dAECM for downstream 3D bioprinting and biological utility, we assessed their compatibility with human umbilical cord venous endothelial cells (HUVECs). Calcein‐AM/EthD‐1 double staining showed that dAECM hydrogels can support HUVECs survival for 5 days with few dead cells (stained red), which implies that dAECM hydrogels are cell‐friendly (Figure 2J).

### 2.3 Design and Characterization of Multicomponent Bioinks for 3D Printing

**Figure 3:**
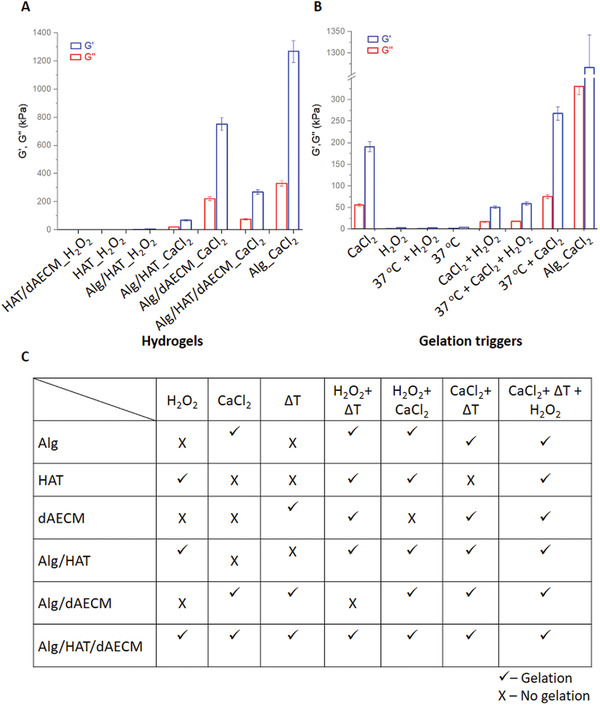
Initial screening for gelation. A) Storage (*G*′) and loss (*G*′) moduli of one‐, two, and multi‐component hydrogels created with CaCl_2_ or H_2_O_2_. B) Combinations of gelation triggers (CaCl_2_, H_2_O_2_, and Δ*T*) to create Alg/HAT/dAECM multicomponent hydrogels with tunable *G*′ and *G*′ values. C) Screening of various molecular components and their combinations for gelation using single or combination of gelation triggers.

In order to prepare suitable multicomponent bioinks with optimum mechanical properties for 3D printing, first we determined the optimum concentrations of individual components to create self‐supporting single‐ and multi‐component hydrogels. Then, we compared the mechanical property of the single‐component hydrogels with their multicomponent counterparts. An aqueous solution of Alg (15% wt) was spontaneously transformed into self‐supporting hydrogel upon the addition of Ca^2+^ ions (0.2 m). On the other hand, an aqueous solution of HAT (4% wt) and HRP (2 U mL^−1^) instantly formed hydrogel when mixed with H_2_O_2_ solution (1 mm). Similarly, we prepared the multicomponent hydrogels as follows: Alg (15% wt)_HAT (4% wt), Alg (15% wt)_dAECM (1% wt), HAT (4% wt)_dAECM (1% wt), and Alg (15% wt)_HAT (4% wt)_dAECM (1% wt) using either Ca^2+^ ions or H_2_O_2_ or both Ca^2+^ ions and H_2_O_2_ as gelation triggers (**Figure** **3**). We also tried various components ratios to create multicomponent hydrogels but could not produce robust hydrogels (Figure S2, Supporting Information).

We performed ATR‐FTIR spectroscopy to confirm successful incorporation of each component into Alg/HAT/dAECM hydrogels. We assigned the broad band at 1600–1700 cm^−1^ to the C=O asymmetric stretching vibrations to Alg carboxylate group, and sharp peak at 1000–1200 cm^−1^ was to C—O—C stretching vibrations in polysaccharide backbone with O‐glycosidic bond (Figure S3, Supporting Information). In the case of HAT, in addition to the peak associating with polysaccharide backbone, the spectrum also reveals peaks at 1600–1700 cm^−1^, 1500–1600, and 1420 cm^−1^ which are attributed to C=O, amide bonds, and C—O vibrational stretching, respectively. The spectrum for Alg/HAT incorporates both the fingerprints of Alg and HAT. The spectrum of dAECM shows distinct amide I and amide II peaks observed at 1500–1700 and 1200–1300 cm^−1^, which confirms the polypeptidic structure of dAECM. With the multicomponent Alg/HAT/dAECM hydrogel, the characteristic peaks of Alg, HAT, and dAECM were observed, which confirmed that the three components were successfully incorporated into the chemical structure of the multicomponent hydrogel.

We carried out dynamic oscillatory rheology to obtain both frequency and amplitude sweep rheographs of the hydrogels. In all cases, both storage (G´) and loss (G´´) moduli are frequency‐independent in the low frequency region and within the linear viscoelastic region, which suggests a gel‐like behavior. The G´ values for Alg and HAT were 126 MPa and 264 Pa, respectively (Figure 3A), suggesting that Alg hydrogels are stiffer than HAT hydrogels by several orders of magnitude. It is important to mention that although Alg hydrogels displayed high G’ value, they are physically brittle. The brittle nature of the hydrogels can be attributed to rapid and inhomogeneous gelation in the presence of Ca^2+^ ions. The *G*′ values for the two‐component hybrid hydrogels of Alg and HAT prepared with either Ca^2+^ ions or H_2_O_2_ or both Ca^2+^ ions and H_2_O_2_ were also measured. As shown in Figure 3B, we established that the G’ values for the two‐component hydrogels can be tuned by tuning the molecular compositions and/or the gelation triggers used and that the stiffness and handability of the two‐component hydrogels were slightly improved over the single‐component hydrogels.

Furthermore, we demonstrate the possibility to create three‐component hydrogels composed of Alg, HAT, and dAECM, which displayed tunable *G*′ depending on the gelation trigger or combination of triggers used. The *G*′ values of the multicomponent hydrogels prepared with H_2_O_2_, ∆*T* (20 °C to 37 °C) and Ca^2+^ ions are 2.74, 4.17, and 191.32 kPa, respectively. Orthogonal gelation using combined gelation triggers ∆*T* + H_2_O_2_, H_2_O_2_ + Ca^2+^, and ∆*T* + Ca^2+^ generate multicomponent hydrogels with G′ values of 2.96, 51.23, and 268.15 kPa, respectively. The tripartite gelation involving ∆*T* + H_2_O_2_ + Ca^2+^ ions generates multicomponent hydrogels with improved *G*′ value of 59.79 kPa (Figure 3B). Although the multicomponent hydrogels prepared with tripartite gelation trigger (∆*T* + H_2_O_2_ + Ca^2+^ ions) display lower *G*′ values compare to the hydrogels prepared with Ca^2+^ ions or ∆*T* + Ca^2+^ ions, they are more flexible and stretchable. Putting together, we establish the possibility to optimize the design of multicomponent hydrogel with potential application as bioink for 3D printing by a careful combination of gelation triggers and molecular compositions (Figure 3C).

It is desirable that Alg/HAT/dAECM hydrogels as bioink have an appropriate shear‐thinning property for an effective 3D printing. For this purpose, we assessed changes in the *G*″ and *G*′ values of Alg, Alg/HAT, and Alg/HAT/dAECM hydrogels as a function of shear strain (%). As shown in the amplitude sweep rheographs, Alg hydrogels display low critical value at 3% (Figure S4, Supporting Information). With the addition of HAT to Alg, the critical strain value (shear strain value at which the materials failed, *G*″ > *G*′) was increased to 10%. Interestingly, the critical strain value was further increased to 15% upon the incorporation of dAECM into the multicomponent hydrogel bioinks. Clearly, the addition of HAT and dAECM to Alg hydrogels enabled the creation of tough multicomponent hydrogels. Moreover, Alg/HAT/dAECM hydrogels exhibited elastic behavior with a shear stress value of ≈30%, suggesting that the multicomponent hydrogels can retain their shapes after 3D printing pressure. Overall, we established that the addition of HAT and dAECM to Alg imparts the multicomponent Alg/HAT/dAECM hydrogels with a significant flow behavior, which is key for extrusion‐based 3D printing.

### 2.4 3D Printing With Multicomponent Hydrogel Bioinks and the Mechanics of Printed Constructs

**Figure 4:**
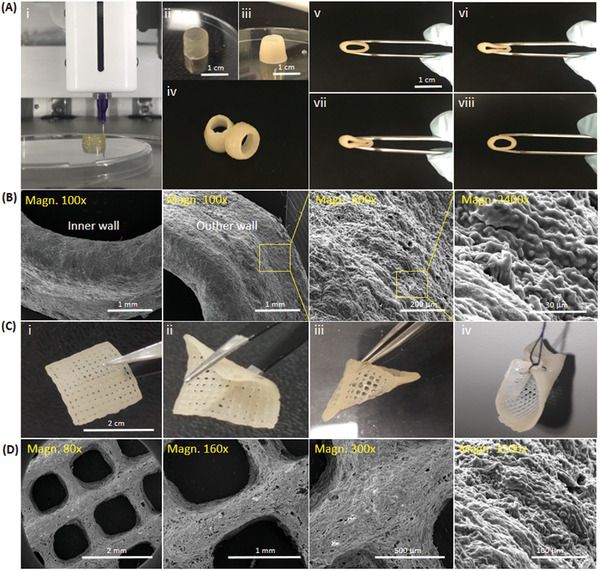
Macroscopic and microscopic analysis of 3D‐bioprinted constructs. A‐i) 3D printing of Alg/HAT/dAECM hydrogels into a tubular vascular construct (diameter: 1 cm). A‐ii) The translucent view of the graft before cross‐linking step A‐iii) turned into an opaque tubular structure upon incubation in an aqueous mixture of H_2_O_2_ and CaCl_2_ at 37 °C. A‐iv) The emerged self‐supporting and mechanically robust scaffolds A‐v–viii)displayed high elasticity and shape‐memory property. B) SEM images showed that the grafts have a fused, homogenous, and round surface morphology in the inner and outer walls. In addition, the fibrous microstructure provided by dAECM is visible at higher magnifications. C‐i–iii)) Hydrogels with different geometry, size, and porosity can be constructed using the developed bioinks. C‐iv)The emerged biomaterials are highly elastic and suturable. D) The constructs obtained in various configurations and sizes exhibit the same fused, homogenous, and fibrous morphology.

We used extrusion printing technique to biofabricate multicomponent Alg/HAT/dAECM hydrogel bioinks into ring geometry to create vascular constructs (**Figure** **4**A‐i). Post‐printing processing of the multicomponent hydrogels with a mixture of CaCl_2_ and H_2_O_2_ transformed the translucent constructs (Figure 4A‐ii) into to an opaque appearance (Figure 4A‐iii), which is suggestive of an improved crosslinking of the printed constructs. The resulting constructs were self‐supporting (Figure 4A‐iv) and regained their initial shapes after manual manipulation by compressing and relaxing (Figure 4A‐v–viii). This interesting phenomenon is crucial for the development of high‐performance constructs for vascular tissue engineering applications.^[^ ^39^ ^]^

We observed microstructure of the obtained constructs using SEM. The low magnification micrographs showed that Alg/HAT/dAECM printed constructs displayed fused, homogenous, and round surface morphology both in the inner and outer walls (Figure 4B). In addition, we observed uniform distribution of nanofibers in the micrographs at higher magnification, which confirms social interactions of dAECM molecular components with the other building blocks in the printed constructs. It is noteworthy that Alg/HAT/dAECM multicomponent bioink can also be utilized to bioprint scaffolds of various grid patterns, shapes, and sizes (Figure 4C‐i). Furthermore, we observed that the printed multicomponent constructs have sufficient robustness and elasticity to withstand various forms of mechanical manipulations such as bending (Figure 4C‐ii) and twisting (Figure 4C‐iii) without apparent damages. In addition, we demonstrated that the constructs can be sutured (Figure 4C‐iv). The multicomponent constructs printed into different grid patterns displayed fused, homogenous, and fibrous morphology similar to the macro‐ and micro‐structures of the printed vascular constructs (Figure 4D). We also printed two‐component hydrogels composed of Alg/HAT, Alg/dAECM, and HAT/dAECM but these bioinks could only create fragile constructs (Figure S5, Supporting Information).

### 2.5 Mechanical Durability, Elasticity, Recovery, and Stability of 3D Printed Alg/HAT/dAECM Multicomponent Constructs

**Figure 5:**
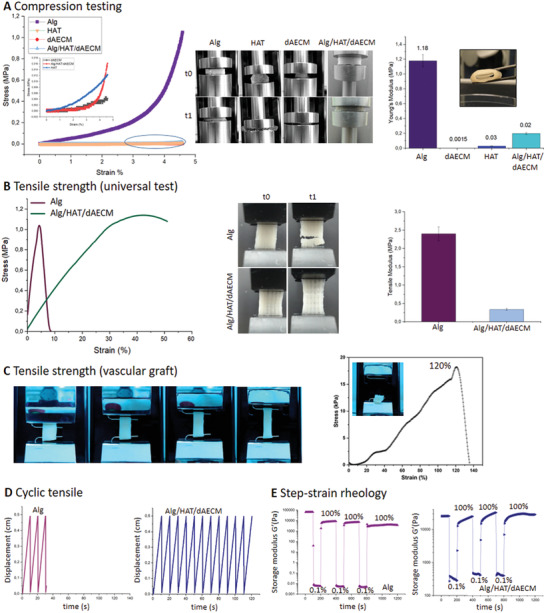
Assessment of robustness and elasticity of Alg/HAT/dAECM multicomponent vascular constructs. A) Compression strength graph comparing the stiffness of single‐component and multi‐component hydrogels. Macroscopic demonstration of compressibility showed that Alg hydrogels are more resistant to compression than the visco‐elastic Alg/HAT/dAECM multicomponent hydrogels. B) Tensile strength curves of Alg and Alg/HAT/dAECM hydrogels (2 × 4 cm). While pure Alg‐based hydrogels were fractured under low stress values and below 5% strain value, Alg/HAT/dAECM multicomponent hydrogels remained intact up to 30% strain, confirming the elastic property of the vascular constructs. C) Tensile strength for 3D printed vascular constructs; can withstand up to 120% strain value. D) Cyclic tensile graphs for Alg and Alg/HAT/dAECM hydrogels further demonstrated the strength of Alg/HAT/dAECM against repetitive deformation. E) Dynamic amplitude measurements supported the elasticity and ability of Alg/HAT/dAECM multicomponent constructs to self‐recover from high shear force.

To further characterize the mechanical properties of 3D printed multicomponent hydrogels prepared with tripartite gelation trigger, we carried out compressive and tensile strength measurements. As shown in **Figure** **5**A, pure Alg hydrogel bioink displayed high compressive strength with Young's modulus value of 1.18 MPa while the Young's modulus value for the three‐component hydrogels was 0.02 MPa, suggesting that Alg hydrogels can resist higher applied compressive forces before failure than Alg/HAT/dAECM hydrogel bioink. Similarly, the higher tensile strength of pure Alg hydrogels than Alg/HAT/dAECM hydrogels confirms that pure Alg hydrogels are more brittle; and hence non‐stretchable, which is evident in its low strain‐at‐failure value (5%) (Figure 5B). Interestingly, the 3D‐printed Alg/HAT/dAECM vascular constructs can withstand high extensibility (elongation at break) up to ≈120% of its original length (Figure 5C; Movie S1, Supporting Information). The observed elongation at break in our all‐natural multicomponent construct is similar to previously reported value for vascular constructs created with dAECM and synthetic polymer polycaprolactone (PCL).^[^ ^40^ ^]^ In addition, we carried out cyclic tensile testing to assess the recovery of the hydrogels after deformation. As shown in Figure 5D, biofabricated Alg hydrogels can only undergo three consecutive loading–unloading tensile cycles before deformation, whereas, the biofabricated Alg/HAT/dAECM multicomponent vascular constructs underwent twelve consecutive loading–unloading tensile cycles without apparent decrease in displacement. The cyclic tensile testing results were also consistent with the step‐strain rheology. As shown in Figure 5E, hydrogels of pure Alg and Alg/HAT/dAECM displayed 70% and 100% recovery, respectively, after deformation. Clearly, it is evident that the multicomponent hydrogels displayed superior mechanical properties, particularly toughness, high stretchability, and excellent fatigue resistance. Although this level of elasticity has been previously reported for 3D printed vascular constructs created with synthetic bioinks,^[^ ^41^, ^42^ ^]^ 3D‐printed constructs based on purely natural bioinks with such high level of mechanical properties are rare. This suggests that the interplay of multiple non‐covalent (ionic, hydrogen bond) and covalent interactions between various molecular components of our multicomponent hydrogel bioink underpins the impressive mechanical properties displayed by our printed constructs.

To assess structural stability of 3D printed multicomponent vascular constructs and understand the molecular mechanisms driving the formation of mechanically robust multicomponent hydrogel bioink, we immersed constructs in an aqueous solution of Ca^2+^ ions chelator ethylenediaminetetraacetic acid (EDTA). As expected, as of day 1 of immersion, the multicomponent vascular constructs became translucent and started to lose their shape fidelity (Figure S6, Supporting Information). In contrast, multicomponent vascular construct immersed in PBS solution retained its texture, shape fidelity, and dimensionality within the same timeframe. This observation suggests that, diffusion of EDTA into the constructs results in Ca^2+^ ions chelation, leading to gradual erosion of Alg and maybe some dAECM components to generate constructs that lack self‐supporting ability. Comparing the swelling property of Alg, Alg/HAT hydrogels and multicomponent vascular construct, we observed that by adding HAT, percentage swelling of Alg increased by 130%, whereas incorporation of dAECM decreased percentage swelling in Alg/HAT/dAECM multicomponent construct by 30% (Figure S7, Supporting Information), suggesting that the multicomponent hydrogel constructs have less porous internal structures compared to Alg/HAT hydrogels, which is consistent with the SEM micrographs (Figure 4B).

Put together, based on the above results, we reasoned that the mechanical properties of the multicomponent hydrogels are not only a function of their molecular compositions but also of the gelation trigger(s) used and the resulting structural networks. For the pure Alg hydrogels, the alginate chains were cross‐linked by strong ionic interaction between the positively charged Ca^2+^ ions and negatively charged alginate chains. The high intramolecular chain crosslinking density makes Alg hydrogels denser, leading to a rigid chain that does not move easily under deformation and causes the hydrogels to break easily. In contrast, the interpenetrated network structures of Alg, HAT, and biomolecular compositions of dAECM crosslinked using orthogonal strategies resulted in the formation of mechanically robust and flexible hydrogels with improved stretchability and printability. Calcium ions complexation with the molecular building blocks of the multicomponent hydrogel bioink is critical for the formation of mechanically robust vascular constructs.

### 2.6 Biological Assessment of Alg/HAT/dAECM Graft

**Figure 6:**
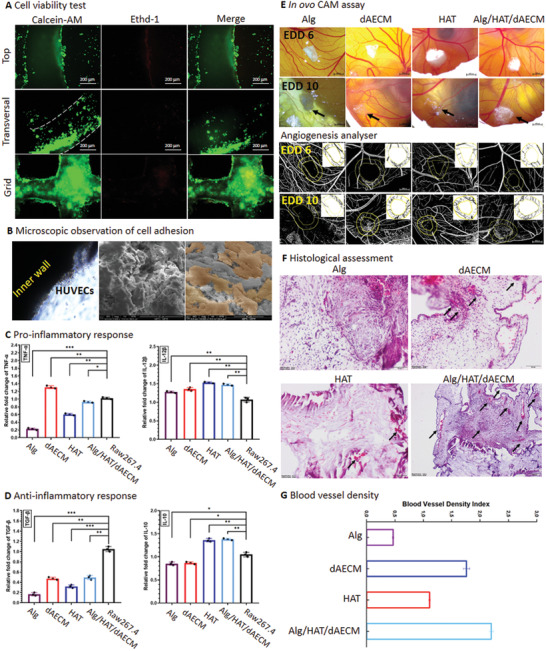
Biological assessment of Alg/HAT/dAECM multicomponent construct. A) Fluorescent microscope images showing the live (stained green) and dead (stained in red) cells. B) Inverted microscope image and SEM image shows HUVECs attachment and adhesion on the inner wall of the vascular constructs. C) Gene expressions of pro‐inflammatory cytokines (TNF‐*α* and IL‐12*β*) in Raw 264.7 macrophages in response to treatment with Alg/HAT/dAECM (*n* = 3, *p > 0.05, **p < 0.01, and *p < 0.001). D) Gene expressions of anti‐inflammatory cytokines (TGF‐*β* and IL‐10) in Raw 264.7 macrophages in response to treatment with Alg/HAT/dAECM (*n* = 3, **p < 0.01, *p < 0.001). E) In ovo CAM assay for Alg, dAECM, HAT, and Alg/HAT/dAECM multicomponent constructs to evaluate the potential of each component as well as the multicomponent constructs to promote blood vessel formation. The images were captured on a stereomicroscope before (EED6) and after (EED10) implantation, and binary images of the whole and designated area (given as inset on the whole image) resulting from further processing with ImageJ Angiogenesis Analyzer. F) Histological assessment of graft integrity and blood vessel formation by H&E staining. G) Bar graph showing the blood vessel density index underneath each explant (*n* = 3).

We first carried out Calcein‐AM/EthD‐1 staining for HUVECs to assess cytocompatibility and cell adhesive properties of printed Alg/HAT/dAECM multicomponent constructs. For this purpose, HUVECs were seeded onto 3D‐printed Alg/HAT/dAECM vascular constructs as well as the grid‐shape Alg/HAT/dAECM multicomponent structures. The cells on both geometries displayed high cell viability and low cell death, implying that Alg/HAT/dAECM constructs are cytocompatible (**Figure** **6**A). In addition, we observed cell migration into the construct when the transverse section of the constructs was examined. In addition, the cells adhered to grid‐shape Alg/HAT/dAECM constructs. By taking a closer look at the spatial distribution of cells on the vascular constructs, we observed a high density of cells adhering to the lumen of the vascular construct (Figure 6B). We used 2,3‐Bis‐(2‐Methoxy‐4‐Nitro‐5‐Sulfophenyl)‐2*H*‐Tetrazolium‐5‐Carboxanilide (XTT) assay to show that the HUVECs seeded on Alg/HAT/dAECM vascular constructs maintained their proliferative capability up to 5 days (Figure S8, Supporting Information). As expected, the cells seeded on pure Alg constructs showed very low cell proliferation whereas incorporation of HAT resulted in a slightly increased cell proliferation. Strikingly, the proliferative capacity of HUVECs was significantly improved upon the incorporation of dAECM in the bioink. We reasoned that the significant improvement in cell proliferation in the presence of dAECM can be attributed to collagen enrichment in the decellularized bioink, which imparts dAECM with cell adhesive epitopes.^[^ ^43^ ^]^

Unregulated inflammatory response to biomaterial implants plays a crucial role in the episode of implant rejection and associated complications. Therefore, we assessed the inflammatory response to Alg/HAT/dAECM multicomponent vascular constructs by measuring the expression levels of the pro‐inflammatory cytokines (TNF‐*α* and IL‐12*β*) and anti‐inflammatory cytokines (TGF‐*β* and IL‐10) in Raw 264.7 macrophages. The gene expression analysis showed that both Alg and HAT can effectively reduce the expressions of pro‐ and anti‐inflammatory cytokines (TNF‐*α* and TGF‐*β*), while dAECM slightly increased the pro‐inflammatory cytokine expressions (Figure 6C,D). A similar observation has previously been reported for an acellular homograft valve in cardiovascular surgery,^[^ ^44^ ^]^ which can be attributed to the residual nucleic acid content in dECMs. More importantly, we revealed that the expression of TNF‐*α* in the Alg/HAT/dAECM‐treated group was slightly reduced by nearly 10%, whereas IL‐10 increased by ≈40%, relative to the control cells (Figure 6C,D). These findings show that the Alg/HAT/dAECM multicomponent vascular constructs displayed anti‐inflammatory properties.^[^ ^45^ ^]^ Alg/HAT/dAECM graft‐mediated pro‐inflammatory response was also confirmed by the downregulated IL‐12*β* and upregulated TGF‐*β* expressions.

To further assess the biological functionality of Alg/HAT/dAECM multicomponent constructs, we performed a preclinical study using chorioallantoic membrane (CAM) assay (Figure 6E). Stereomicroscopic images revealed that hydrogels of dAECM and HAT were largely disintegrated in the CAM due to their relatively weak mechanical strength, whereas Alg and Alg/HAT/dAECM retained their bulk structures after 5 days in the CAM. After 5 days of incubation, we analyzed the images of CAM underneath hydrogel constructs using Image J Angiogenesis Analyzer (Figure 6E). In addition, we assessed blood vessel formation and graft structural integrity using H&E staining. The histograms confirmed the stereomicroscopic observations and demonstrated that Alg/HAT/dAECM multicomponent constructs preserved their structural integrity during implantation while significantly promoting blood vessels formation (Figure 6E,F). We also calculated the blood vessel density index for the newly formed vessels. The index of Alg/HAT/dAECM was found to be higher than those of Alg, dAECM, and HAT by 4.5‐, 1.25‐, and 2‐folds, respectively (Figure 6G). A previously published work established that Alg has no angio‐inductive potential.^[^ ^25^ ^]^ Therefore, we reasoned that HAT and dAECM are the primary components of our multicomponent constructs that promote blood vessels formation.

### 2.7 Pre‐Surgical Demonstrations: Perfusion and Leak Test

**Figure 7:**
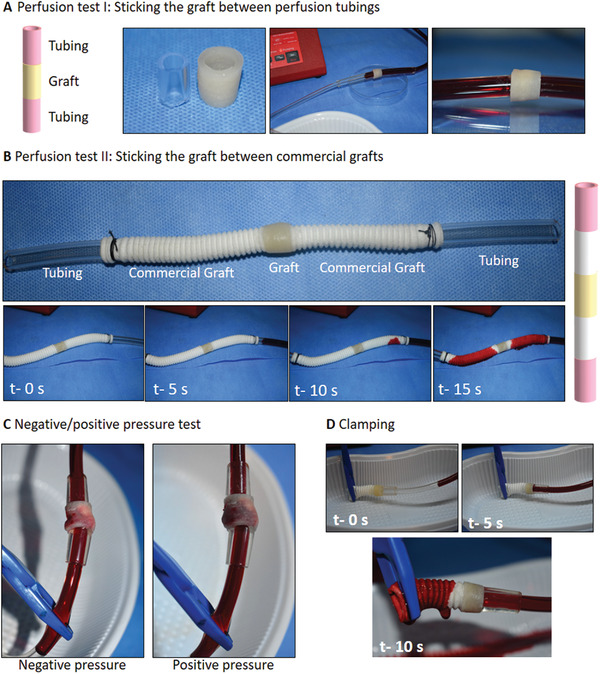
Surgical pre‐applications of 3D printed Alg/HAT/dAECM vascular graft. A) Demonstration of the vascular graft engrafted between perfusion tubes. B) Demonstration of cardiovascular application of 3D printed Alg/HAT/dAECM vascular graft that was engrafted between a commercial graft. C) Inflation test of 3D printed Alg/HAT/dAECM vascular graft via negative/positive pressure application. D) 3D printed Alg/HAT/dAECM vascular graft inserted between a perfusion tubing and clamped commercial graft.

To demonstrate the practical applicability of Alg/HAT/dAECM multicomponent vascular constructs, we assessed their potential utility in several ways. To recreate the tubular geometry (diameter = 1.3 cm) and average blood flow rate in the mouse aorta (11.4 mL min^−1^), respectively,^[^ ^46^ ^]^ first, we grafted our 3D bioprinted constructs (*d* = 1.3 cm) between two polyvinylchloride tubes using a commercially available tissue glue and perfused with a Harvard's apparatus at a flow rate of 10 mL min^−1^ (**Figure** **7**A). The multicomponent vascular constructs retained their shape integrity and structural stability after 1 h of perfusion with no evidence of leakage (Movie S2, Supporting Information). To further demonstrate the potential of the multicomponent vascular constructs for clinical use, we made an incision on a piece of commercial vascular graft totally prepared from non‐biological polymers and engrafted the printed Alg/HAT/dAECM multicomponent vascular construct (Figure 7B). Both ends of the commercial grafts were stitched to the perfusion tubing. Although we observed fluid leakage through the commercial graft when perfused with red dye, our multicomponent vascular construct prevented leakage and retained its durability after 1 h of perfusion (Movie S3, Supporting Information). Further, we adhered the graft between two perfusion tubes using commercially available tissue glue; and then, tested the response of Alg/HAT/dAECM multicomponent vascular construct to both negative and positive pressure cycles (ten times) (Figure 7C; Movie S4, Supporting Information). The multicomponent vascular construct was compressed under negative pressure while it was expanded when exposed to high positive pressure. Last, we showed that the multicomponent vascular construct can withstand inflation when adhered between a perfusion tube and another commercial graft (Figure 7D). According to Gong et al. (2016), an ideal vascular graft should exhibit high biocompatibility, resistance to high liquid pressure, and display elastic properties reminiscent of native vascular tissues.^[^ ^47^ ^]^ In the light of these material design requirements, our 3D bioprinted Alg/HAT/dAECM multicomponent vascular constructs hold great potential for real‐life clinical use for vascular tissue engineering.

## 3 Conclusion

3D printing of functional synthetic analogues of the native tissues is an evolving field and our results presented herein demonstrate the possibility to extend the frontiers of biofabrication and tissue engineering using a multicomponent bioink design strategy that uses purely naturally derived building blocks. Here, we developed a three‐component bioink composed of alginate, hyaluronic acid, and decellularized extracellular matrix to create mechanically robust, elastic, and bioactive 3D‐printed constructs. The multicomponent bioink was printed into aorta vascular grafts that showed excellent hydrodynamic performance, cyclic compressive property and shape fidelity. The printed constructs could promote angiogenesis in vitro and in vivo, as well as, anti‐inflammatory responses. This thus suggests their potential application in cardiovascular surgery. Furthermore, our technology is highly adaptable and can be tuned to create highly elastic and functional tissue analogues for other types of tissue engineering applications by using the tissue of interest in the decellularization process. Considering the simplicity of the bioink preparation, the multicomponent bioink design strategy holds great promise for clinical applications in skin and connective tissue engineering.

## 4 Experimental Section

### Aorta Decellularization and Characterization of dAECM and its Hydrogel

*Aorta Decellularization and Digestion*: Bovine aorta tissues were mechanically dissected from the whole heart, and the remaining pieces of cardiac and fat tissue were removed. The hearts were purchased from a local slaughterhouse and no ethical approval was needed. The specimens were cut into 1 cm × 5 cm pieces and thoroughly washed with ultrapure water (Merck–Millipore, Germany). Following a treatment step with EDTA (0.1% wt) in Tris buffer (10 mm, pH = 8.0) in hypotonic condition, aorta tissues were decellularized by SDS (1% wt) and DMSO (1 m) for 3 h at 37 °C. Nuclear materials were removed from the decellularized tissues through digestion with DNase I (200 µg mL^−1^, ≥400 Kunitz unit per mg protein, Sigma, USA) and RNase A (50 µg mL^−1^, 50–100 Kunitz unit per mg protein, Sigma, USA), MgCl_2_ (10 mm), and Trizma hydrochloride (50 mm). dAECM was washed with DMEM media supplemented with antibiotic/antimycotic (3%, v/v) for 2 days to decontaminate and remove the remnant DMSO. After a further washing step with PBS, the tissues were homogenized (T‐18 Basic Ultra TURRAX, Germany) and freeze‐dried (Telstar, LyoQuest, Spain). To prepare pre‐gel and hydrogel, the dAECM was cut into small pieces, weighed to be 10 mg mL^−1^, and digested in 0.01 m HCl including pepsin (600 U mg^−1^) for 72 h at RT under rotation. Pre‐hydrogel (not neutralized) was then centrifuged at 14 000 rpm for 5 min to remove undigested particles. To obtain dAECM hydrogel, pre‐hydrogel was neutralized with sodium bicarbonate (7.5% wt) and incubated at 37 °C for 1 h to induce the fibrillation process.

*Double Stranded DNA (DsDNA) and Sulfated Gag Content Analyses*: dsDNA was isolated via a genomic DNA purification kit (Thermo Scientific, GeneJET, USA), and quantification of dsDNA was performed as previously described.^[^ ^48^ ^]^ The concentration of sGAGs was quantified using dimethyl methylene blue dye‐binding assay as previously described.^[^ ^48^ ^]^ Last, the BCA assay (Thermo Scientific, USA) was handled to determine the final concentration of dAECMs through spectroscopical (562 nm) measurements.^[^ ^48^ ^]^ The spectroscopic measurements were carried out by a Multiscan GO µdrop micro‐plate reader (Thermo Scientific, USA).

*Histology*: Structural analysis of native aorta and dAECM was examined through a histological study. For this purpose, native and decellularized tissues were fixed in paraformaldehyde (%10, v/v) for 24 h at 4 ^°^C and then dehydrated in ethanol series. The tissues were then embedded in paraffin, sectioned (3–5 µm), and after deparaffinization with xylene, the sections were stained with H&E and Masson's Trichorme.

*Proteomics Analysis*: Proteins were precipitated with acetone and dissolved in 100 mm ammonium bicarbonate containing 20% methanol. 100 µg of protein was reduced with dithiothreitol (DTT, 10 mm) for 15 min at 56 °C and alkylated with iodoacetamide (IAA, 55 mm) for 30 min at room temperature in the dark. After the cleaning protocol, proteins were digested with trypsin (1:100, w:w) and incubated overnight. Formic acid was added to the solution to stop enzymatic digestion. Analysis was performed by LC‐qTOF‐MS and peptides were run on a C18 column (Agilent, 100 × 1 mm, 3.5 µm) with buffer A (0.1% formic acid in water) and eluted with a 145 min gradient time, with 2% to 90% buffer B (0.1% FA in acetonitrile) at a flow rate of 0.15 mL min^−1^. The scan range was set to 300–1700 m/z in positive ionization MS/MS mode. Peptide fragmentation was performed via the following collision energy formula; slope: 3.6 and offset: −4.8. MS/MS data were analyzed using the computational proteomic analysis software with the MaxQuant (Max Planck Institute of Biochemistry). The smallest possible peptide length was determined as 6 and the longest peptide length was determined as 40 amino acids during the identification of the peptides. Proteins were identified using the UniProt Human Database. The lowest score value of the modified peptides was set as 10. Statistical analysis was performed with Perseus. In the Perseus platform, the reverse, potential, and only identified by site proteins were filtered from the data matrix. The LFQ values of the proteins were transformed to Log 2. For each sample, it was preferred that the proteins be quantified in at least two technical replicates, and unquantifiable proteins were excluded from the data.

*Rheology*: Rheological measurement of dAECM hydrogel (1% wt) was performed using a Discovery Hybrid Rheometer (TA Instruments, USA) with parallel plates of cross‐hatched geometry (*φ*=40 mm, gap 0.9 ± 0.1 mm). Time sweep rheology was performed for dAECM hydrogel between at 1 Hz and 37 °C.

*Cell Viability Assay*: HUVECs were obtained from Merk (USA), grown in DMEM‐HG supplemented with 10% fetal bovine serum (FBS) and 1% penicillin–streptomycin (P/S) (Thermo Fisher, USA) and were incubated in incubator conditions at 37 °C in humidified air with 5% CO_2_. The medium was changed every 2–3 days with fresh medium until the desired cell confluence was obtained. Before cell seeding on hydrogels, 0.25% trypsin‐EDTA was used for dissociation of the cells. Live/dead testing was proceeded to examine biocompatibility of the dAECM. For this purpose, dAECM (1% wt) was neutralized with PBS (10×) and NaOH (1 N) in 96‐well plate. Neutralized dAECMs were incubated 45 min at 37 °C to achieve successful hydrogelation. Before cell seeding, dAECM hydrogels were sterilized by washing with DPBS supplemented with P/S (1%) and then DPBS residues were removed by washing with cell culture medium. HUVECs (20 000 cells per gel) were seeded on hydrogels and cultured for up to 5 days. For qualification of cell survival, fluorescence staining was evaluated for live cells with Calcein‐AM (4 µm, Molecular Probes, Thermo Fisher, UK) and dead cells with EthD‐1 (2 µm, Molecular Probes, Thermo Fisher, UK). Cells were visualized under the fluorescent microscope (Leica DMIL, Germany) at 488 nm wavelength for Calcein‐AM and 527 nm for EthD‐1.

### Development and Rheological Characterization of Multicomponent Bioink

*Development of Bioinks*: dAECM (1% wt) hydrogel was prepared by neutralizing with 10× PBS (1:9 v/v) following the introduction of NaOH (1N, 1:10 v/v) and was incubated 45 min at 37 °C to achieve successful hydrogelation. Alginate (15% wt, Sigma) was dissolved in DPBS (Merk, USA) and CaCl_2_ (0.2 m, 1:5 v/v) was introduced for ionic crosslinking. Last, synthesis of HAT was based on the amidation of the carboxylic acid groups on HA with amine groups on tyramine, as previously described and fully characterized.^[^ ^49^ ^]^ HAT hydrogels were prepared by first dissolving HAT (4% w/v) in DPBS containing HRP (2 U mL^−1^, Sigma–Aldrich, USA); then, gelation was triggered by adding H_2_O_2_ (2 mm). Bioink combinations of Alg/HAT, Alg/dAECM, dAECM/HAT, and Alg/HAT/dAECM were prepared for further experiments. Alg (15% wt)/HAT(4% wt) was prepared by combining Alg and HAT solutions in 1.5:1 (v/v) ratio and crosslinking was driven by adding H_2_O_2_ (2 mm) and CaCl_2_ (0.2 m, 1:5 v/v), respectively. In the case of dAECM‐including formulations such as Alg (15% wt)/dAECM(1% wt) in 1.5:1 (v/v) ratio and HTA(4% wt)/dAECM(1% wt) 1:1 (v/v) ratio, pre‐neutralization of dAECM was achieved by introducing neutralizing agents and incubating for 15 min at 37 °C. Then, Alg or HAT solution was mixed with the pre‐neutralized dAECM and complete neutralization was proceeded by incubating formulations at 37 °C for 45 min. Hydrogelation of two‐component scaffolds was carried out by using mixtures of gelation triggers and biopolymers as follows: Alg (CaCl_2_, 0.2 m, 1/5 v/v) and HAT (H_2_O_2_, 2 mm, 1/10 v/v). Last, 3D‐printable, highly elastic, and bioactive formulation was developed by combining Alg (15% wt), HTA(4% wt), and dAECM(1% wt) solutions in 1.5:1:1 (v/v/v) ratio and homogenization by thorough mixing. Hydrogelation was achieved by orthogonal enzymatic (H_2_O_2_, 2 mm), ionic (CaCl_2_, 0.2 m, 1/5 v/v), and thermal (37 °C) processes.

*Rheology*: The rheological properties of the hydrogels of all hydrogels were characterized on a DHR‐2 Rheometer (TA Instruments, USA). Hydrogels were placed between 40 mm cross‐hatched parallel plates separated by a 1 mm gap. First, an oscillatory amplitude sweep was performed at a frequency of 1 Hz and a strain range of 0.01–100%. The strain amplitude value was found to be 0.15%. Then, an oscillatory frequency sweep was performed in the range of 0.1–100 Hz under the constant strain of 0.15% to assess the behavior of the gel. All the rheological measurements were carried out at 25 °C.

### 3D Printing of Vascular Graft Using the Multicomponent Hydrogel

For 3D printing, Alg and Alg/HAT/dAECM hydrogels bioink were prepared by mixing aqueous solutions of Alg (15% wt), HTA(4% wt), and dAECM(1% wt) in 1.5:1:1 volumetric ratio and mixing thoroughly. Hydrogel scaffolds were created using customized three‐axis Axo C2 3D‐printer machine mounted with a printing head. G‐codes were designed using Phyton, imported to the open source software Repetier‐HostV2.1.6 program to control the three‐axis translational platform. To optimize the parameters of printability, bioinks were printed through 21G, 23G, and 34G nozzle; the printing pressure was altered to 30, 40, 50, and 55 kPa; and the pore sizes of the grid geometry of 20%, 25%, and 30% fill density were tested. After optimization and for the further experiments, the bioinks were transferred into a 3 mL syringe reservoir modified with a 23G nozzle (Axolotl Biosystems, Turkey). The axis parameters of printed constructs were designed as *x*:0.6 cm, *y*:0,6 cm, and *z*:0.5 cm with 25% fill density for grid geometry and *x*:1.3 cm, *y*:1.3 cm, *z*:1.5 cm with 0% fill for vessel geometry. Printing was proceeded at 55 kPa pressure and 25 °C tray temperature. After the 3D printing was completed, Alg was crosslinked by covering scaffold with CaCl_2_ (0.2 m) solution while, Alg/HAT/dAECM was covered with crosslinker mixture containing H_2_O_2_ (20 mm)/CaCl_2_(0.2 m) (1:10 v/v) and incubated for 2 h at 37 °C for highly elastic hydrogel scaffold.

### Mechanical and Microstructural Characterization of 3D Printed Vascular Graft

*Compression and Tensile Tests*: Uniaxial compression tests of prepared samples were conducted using a micromechanical testing device (UniVert, CellScale Biomaterials Testing) with a 50 N load cell. HAT (4% wt, cylindrical, *ø* = 13.06 mm; *h* = 9 mm), dAECM (1% wt, cylindrical, *ø* = 11.4 mm; *h* = 5 mm), and Alg (15% wt., length: 12 mm, width: 10, h: 5 mm) hydrogels were mounted between the two self‐leveling plates and compressed at a rate of 0.05 mm s^−1^ until the compression ratio was ≈85–90%. In addition, a uniaxial tensile test was also performed for the 3D‐printed vascular graft consisting of Alg/HAT/dAECM composite hydrogel (cylindrical, *ø* = 12 mm; *h* = 15 mm). A cylindrical vascular graft was placed in a paper clip, and both ends exposed to the paper clip were fixed between the two jaws of the device. The tensile stiffness of the sample was investigated by applying a strain rate of 0.3 mm s^−1^ until breaking occurred. The real‐time images were taken with a tripod camera (HD 1080p, Logitech) at a frequency of 5 Hz during all measurements. The force and displacement data were processed to analyze the samples' mechanical features, and stress–strain curves were achieved. To further assess the ability of hydrogel to recover after a tensile stress, tensile cyclic loading–unloading tests were also applied for 120 s up to 25% strain.

*Dynamic Oscillatory Rheology*: The recovery ability of the hydrogels in response to applied shear forces was additionally examined using the following procedure: 0.1% (100 s), 100% (200 s), 0.1% (100 s), 100% (200 s), 0.1% (100 s), and 100% (400 s) with the applied shear force.^[^ ^50^ ^]^

*Scanning Electron Microscopy*: The microstructures of 3D‐printed constructs were characterized using SEM. Constructs were processed for SEM with freeze‐dryer to remove the water and other solvents. The constructs were washed, frosted overnight, freeze‐dried for 1d, sputter‐coated with gold at 20 mA for 45 s, and monitored under SEM (FEI 430 Nova NanoSEM, USA).

### Biological Assessments

*XTT and Cell Viability*: Live/dead and XTT (Biological Industries, USA) testings were performed to examine biocompatibility of the fabricated construct in terms of cell survival and proliferation. For live/dead assay, multi‐component Alg/HAT/dAECM bioink was prepared, followed by 3D printing process in grid and vessel geometry. Hydrogelation was proceeded by incubating in trigger mixtures (H_2_O_2_/CaCl_2_) for 2 h. In advance of cell culture, sterilization of 3D‐printed constructs was achieved by incubating scaffolds in DPBS supplemented with P/S and DPBS residues were removed by washing with cell culture medium. Constructs were placed in 24‐well plate and HUVECs (50 000 cell per gel) were cultured on hydrogel‐constructs for 5 days. At the end of the incubation period, cells were stained with Calcein‐AM/EthD‐1 as previously described and cell viability was assessed under a fluorescent microscope. To further analyze the cell proliferation on multi‐component hydrogel, XTT test was performed. Alg, Alg/HAT, and Alg/HAT/dAECM hydrogels were prepared in 48‐well plate and HUVECs (30 000 cells per gel) were cultured on hydrogels for 3 and 5 days. At the end of the culture periods, washed hydrogels were treated with XTT reagent for 4 h and quantification was performed by recording absorbance values at 490 nm with a microplate spectrophotometer (Multiskan Sky, Thermo Fisher, USA). The statistical difference between two and more than two groups were investigated by *t*‐test and one‐way ANOVA, respectively, followed by Tukey's posthoc test.

*Investigating the Immune Response of Macrophages Against Multicomponent Hydrogel*: Immune‐response of multi‐component hydrogel was investigated by reverse transcriptase‐quantitative polymerase chain reaction (RT‐qPCR). For this purpose, Alg/HAT/dAECM and components of multi‐component bioink (Alg, dAECM, HAT) were prepared in 48‐well plate. Culture medium was introduced to cell‐free hydrogels and 24 h incubation was maintained for pre‐dissociation of hydrogels in culture medium. Simultaneously, Raw267.4 cells (200 000 cells per well) were cultured in 48‐well plates until the end of the incubation period of hydrogels. After 24 h, hydrogels cultured in the medium were dissociated and homogenized by continuous pipetting and final mixtures were centrifuged at 1000 rpm for 2 min for the removal of excess residues. The hydrogel supernatants were collected, and the medium of cultured RAW267.4 cells was replaced with supernatants collected from the hydrogels. At the end of the 3 days’ culture period, total RNA was extracted from cells by utilizing a total RNA isolation kit (GeneDireX, USA). Reverse transcripts were obtained by an iScript cDNA Synthesis Kit (Bio‐Rad, USA) according to the manufacturer's instruction. RT‐qPCR was performed using a thermal cycler (Bio‐Rad CFX96 instrument, USA). Inflammatory response of cells was investigated by measuring the expression levels of the pro‐inflammatory cytokines (TNF‐*α* and IL‐12 *β*) and anti‐inflammatory cytokines (TGF‐*β* and IL‐10). The statistical difference was investigated by *t*‐test and one‐way ANOVA.

*In Ovo Chick Chorioallantoic Membrane (CAM) Assay*: Chicken in ovo CAM assay was conducted to investigate materials’ angiogenic potential. In brief, fertilized chicken eggs were purchased from a local breeder (Çanakkale, Turkey); dirt, feathers, and excrement were gently removed from the eggshells and disinfected with a 20% ethanol‐sprayed paper towel. The eggs were placed in an incubator with forced‐drafted constant humidity (Brinsea Ovation 28 Advance Ex, U.K.) and incubated in position vertically at 37.5 °C and 60% relative humidity (RH) while rotating every 120 min. At embryonic development day 3 (indicated as EDD 3), the shell on the top of the eggs was cut off and opened a window and this aperture was closed with a sterile parafilm. Incubation of embryos was continued until EDD 6 in a still draft incubator at the same culture conditions (60% RH; 37.5°C). On EDD 6, the pre‐sterilized scaffolds (Alg, dAECM, HAT, Alg/HAT/dAECM) were carefully placed between two large vessels on the CAM surface under a stereomicroscope (Stemi 305, Zeiss) with a digital camera (Axiocam 105 color, Zeiss). Macroscopic images of the implants were taken on EDD 6 and 4 days after incubation (EDD 10). Vascular density defined as the percentage area occupied by blood vessels was calculated from the streomicroscopic images of each hydrogel using the ImageJ software containing Vessel Analysis and Vascular Density plugins (ImageJ 1.51j8, National Institutes of Health, Bethesda, MD, USA). To this end, as applied in previous studies,^[^ ^51^, ^52^, ^53^ ^]^ stereomicroscopic images were first converted to binary images using an automated thresholding algorithm; and then, the vascular density was calculated by considering all discernible blood vessels, capillaries, arterioles, and venules in a 1 mm annulus about the imaginary boundary drawn around the scaffolds (Figure 6E). The angiogenic response of the prepared scaffolds was evaluated using the blood vascular density index, which measured the ratio of vascular density in the designated area of the scaffolds at EDD 10 and EDD 6. After the imaging of the implants on EDD 10, in ovo CAM assay was terminated. The CAM–biomaterial complex was excised by leaving a margin of 1 cm CAM around the scaffold and this complex was fixed in 10% formalin for histological evaluations. The histological sectioning and staining for H&E were performed as previously described.

## Conflict of Interest

The authors declare no conflict of interest.

## Supporting information

Supporting Information

Supplemental Movie 1

Supplemental Movie 2

Supplemental Movie 3

Supplemental Movie 4

## Data Availability

The data that support the findings of this study are available from the corresponding author upon reasonable request.
