# Supplementary material for: 3D Printing of Extracellular Matrix‐Based Multicomponent, All‐Natural, Highly Elastic, and Functional Materials toward Vascular Tissue Engineering
Source: Adv Healthc Mater. 2023 Apr 25;12(20):2203044. doi: 10.1002/adhm.202203044 (PMC11468991; doi:10.1002/adhm.202203044)
Supplement: Supplementary file 1 — Supporting Information [file ADHM-12-2203044-s003.pdf]

ADVANCED HEALTHCARE MATERIALS

Supporting Information

for *Adv. Healthcare Mater.*, DOI 10.1002/adhm.202203044

3D Printing of Extracellular Matrix-Based Multicomponent, All-Natural, Highly Elastic, and Functional Materials toward Vascular Tissue Engineering

Melis Isik, Ece Karakaya, Tugba Sezgin Arslan, Deniz Atila, Yasar Kemal Erdogan, Yavuz Emre Arslan, Hakan Eskizengin, Cemil Can Eylem, Emirhan Nemutlu, Batur Ercan, Matteo D'Este, Babatunde O. Okesola and Burak Derkus**

3D printing of Extracellular Matrix-Based Multicomponent, All-Natural, Highly Elastic, and Functional Materials Towards Vascular Tissue Engineering

Melis Isik[1], Ece Karakaya[1], Tugba Sezgin Arslan[1], Deniz Atila[2,3], Yasar Kemal Erdogan[4,5], Yavuz Emre Arslan[6], Hakan Eskizengin[7], Cemil Can Eylem[8], Emirhan Nemutlu[8,9], Batur Ercan[4,10], Matteo D'Este[11], Babatunde Okesola[12*], and Burak Derkus[1*]

1. Stem Cell Research Lab, Department of Chemistry, Faculty of Science, Ankara University, Ankara 06560, Turkey
2. Department of Engineering Sciences, Middle East Technical University, Ankara 06800, Turkey
3. International Centre for Research on Innovative Bio-based Materials (ICRI-BioM), Lodz University of Technology, Lodz, 90924, Poland
4. Biomedical Engineering Program, Middle East Technical University, Ankara 06800, Turkey
5. Department of Biomedical Engineering, Isparta University of Applied Science, Isparta 32260, Turkey
6. Department of Bioengineering, Faculty of Science, Canakkale Onsekiz Mart University, Canakkale, Turkey
7. Department of Biology, Faculty of Science, Ankara University, Ankara 06560, Turkey
8. Analytical Chemistry Division, Faculty of Pharmacy, Hacettepe University, 06230 Ankara, Turkey
9. Bioanalytic and Omics Laboratory, Faculty of Pharmacy, Hacettepe University, Ankara, Turkey
10. Department of Metallurgical and Materials Engineering, Middle East Technical University, Ankara 06800, Turkey
11. AO Research Institute Davos, Clavadelerstrasse 8, Davos Platz 7270, Switzerland
12. School of Life Sciences, University of Nottingham, Nottingham NG7 2UH, UK

*Corresponding Author: bderkus@ankara.edu.tr
*Corresponding Author: babatunde.okesola@nottingham.ac.uk

Supporting Information

1. Proteomics analysis of the decellularized aorta extracellular matrix

Table S1. Identified proteins by LC-qTOF-MS

Protein	Gene	Sample 1	Sample 2	Sample 3	RSD %
2-oxoisovalerate dehydrogenase subunit alpha, mitochondrial	BCKDHA	10.396	10.617	11.107	3.400
5-nucleotidase domain-containing protein 2	NT5DC2	15.368	14.811	15.457	2.303
60S ribosomal protein L14	RPL14	10.937	10.402	11.723	6.029
Acetyl-coenzyme A synthetase, cytoplasmic	ACSS2	13.161	12.316	14.601	8.652
Actin, alpha cardiac muscle 1	ACTC1	11.174	10.680	10.313	4.031
Actin, aortic smooth muscle	ACTA2	18.141	18.102	17.949	0.561
Actin, cytoplasmic 2	ACTG1	14.967	14.988	14.732	0.953
Adipogenesis regulatory factor	ADIRF	10.886	10.977	10.831	0.674
ADP-ribosylation factor 3	ARF3	11.725	12.431	12.550	3.643
Alpha-actinin-1	ACTN1	11.472	12.978	13.065	7.165
Alpha-parvin	PARVA	11.538	11.992	12.610	4.466
Ankyrin repeat domain-containing protein 20B	ANKRD20A 8P	11.076	12.137	12.227	5.420
Ankyrin repeat domain-containing protein 40	ANKRD40	11.073	12.297	11.354	5.535
Ankyrin repeat domain-containing protein 62	ANKRD62	10.773	10.354	11.203	3.939
Annexin A2	ANXA2	11.406	11.955	10.536	6.333
Annexin A5	ANXA5	11.540	11.100	12.179	4.676
Apolipoprotein E	APOE	11.320	11.582	11.107	2.096
Apolipoprotein(a)	LPA	15.081	11.134	11.263	17.94 9
ARF GTPase-activating protein GIT1	GIT1	11.363	13.888	16.682	19.03 3
ATP-binding cassette sub-family A member 7	ABCA7	12.733	10.970	14.066	12.33 3
ATP-binding cassette sub-family C member 2	ABCC2	11.550	10.995	12.355	5.877
ATP-binding cassette sub-family C member 5	ABCC5	11.082	10.731	11.696	4.374
AT-rich interactive domain-containing protein 4B	ARID4B	16.579	15.907	17.109	3.643
Bactericidal permeability-increasing protein	BPI	14.756	19.052	19.021	14.03 6
B-cell CLL/lymphoma 9 protein	BCL9	11.279	10.634	11.138	3.078
Beta-galactoside alpha-2,6-sialyltransferase 1	ST6GAL1	17.500	17.896	17.803	1.168
Biglycan	BGN	11.587	10.353	10.300	6.776
Biorientation of chromosomes in cell division protein 1-like 1	BOD1L1	10.443	10.856	10.653	1.938
Brefeldin A-inhibited guanine nucleotide-exchange protein 1	ARFGEF1	11.496	11.360	11.326	0.787
Cadherin EGF LAG seven-pass G-type receptor 1	CELSR1	12.086	9.969	10.774	9.763
Calcineurin B homologous protein 3	TESC	11.214	13.495	15.366	15.56 5
Calcium homeostasis modulator protein 4	FAM26D	12.663	9.551	14.270	19.73 1
Calponin-1	CNN1	12.824	13.833	13.867	4.386
Carbohydrate deacetylase	YDJC	14.661	12.371	14.538	9.298

CCR4-NOT transcription complex subunit 1	CNOT1	13.235	13.378	13.375	0.611
Centromere protein F	CENPF	10.717	11.762	11.703	5.153
Centrosomal protein of 70 kDa	CEP70	11.509	11.270	11.334	1.090
Centrosome-associated protein 350	CEP350	10.091	10.411	11.287	5.841
CEP295 N-terminal-like protein	KIAA1731N L	15.966	13.103	15.358	10.18 6
Chromodomain-helicase-DNA-binding protein 2	CHD2	11.527	10.432	10.872	5.035
Chromodomain-helicase-DNA-binding protein 3	CHD3	10.854	10.281	10.859	3.115
Cilia- and flagella-associated protein 100	CCDC37	10.804	11.797	11.882	5.214
Claudin-8	CLDN8	12.461	10.539	11.299	8.465
Cleavage stimulation factor subunit 3	CSTF3	10.508	11.173	11.812	5.843
CLIP-associating protein 1	CLASP1	14.945	11.057	18.740	25.75 9
Coagulation factor V	F5	15.189	15.298	15.215	0.371
Collagen alpha-1(I) chain	COL1A1	20.445	20.446	20.265	0.513
Collagen alpha-1(II) chain	COL2A1	10.867	11.924	11.340	4.653
Collagen alpha-1(III) chain	COL3A1	13.398	13.460	13.322	0.519
Collagen alpha-2(I) chain	COL1A2	19.108	19.060	18.937	0.465
Collagen alpha-2(V) chain	COL5A2	16.203	16.723	16.340	1.640
Collagen alpha-2(VI) chain	COL6A2	14.909	14.876	14.834	0.254
Collagen alpha-3(IV) chain	COL4A3	10.757	10.825	11.761	5.047
Collagen alpha-3(VI) chain	COL6A3	18.294	18.278	18.272	0.063
Core-binding factor subunit beta	CBFB	11.471	11.162	10.149	6.328
Cysteine and glycine-rich protein 1	CSRP1	16.548	16.453	16.234	0.981
Cytoplasmic tyrosine-protein kinase BMX	BMX	11.000	11.133	12.591	7.628
Cytoskeleton-associated protein 5	CKAP5	13.905	12.842	13.650	4.122
DENN domain-containing protein 2A	DENND2A	10.640	10.733	10.624	0.549
Desmin	DES	11.937	10.686	10.998	5.808
Destrin	DSTN	14.339	14.399	13.703	2.724
DNA polymerase kappa	POLK	17.653	16.722	15.275	7.242
DNA repair protein SWI5 homolog	SWI5	13.561	13.511	16.768	12.77 1
Double-stranded RNA-binding protein Staufen homolog 1	STAU1	10.194	11.194	11.182	5.288
Dual specificity protein phosphatase 16	DUSP16	11.151	10.038	11.133	5.919
Dual specificity testis-specific protein kinase 1	TESK1	11.160	11.462	10.942	2.332
EF-hand domain-containing family member B	EFHB	10.579	16.568	16.661	23.86 4
EGF-containing fibulin-like extracellular matrix protein 1	EFEMP1	11.149	10.874	11.076	1.295
Elongation factor 1-alpha 1	EEF1A1	13.784	13.543	13.688	0.888
Endoplasmic reticulum lectin 1	ERLEC1	10.808	10.271	10.633	2.587
Eukaryotic peptide chain release factor GTP-binding subunit ERF3A	GSPT1	18.243	10.995	14.060	25.21 2
Eukaryotic translation initiation factor 4B	EIF4B	18.541	18.465	18.185	1.019
Fatty acid-binding protein 12	FABP12	10.151	9.894	11.944	10.47 4
Fatty-acid amide hydrolase 2	FAAH2	10.857	10.956	10.726	1.065

F-box only protein 27	FBXO27	12.751	12.126	12.809	3.012
Fibrillin-1	FBN1	17.409	17.259	17.230	0.555
Fibrillin-3	FBN3	11.367	10.731	11.206	2.978
Fibroblast growth factor receptor 2	FGFR2	14.124	16.235	13.726	9.177
Fibulin-5	FBLN5	14.050	14.068	14.119	0.254
Filamin-A	FLNA	15.299	15.399	15.232	0.550
Filamin-B	FLNB	12.645	12.530	12.434	0.842
Four and a half LIM domains protein 1	FHL1	13.143	14.894	14.787	6.876
Galectin-1	LGALS1	11.996	13.325	11.752	6.853
Gap junction alpha-8 protein	GJA8	16.852	16.828	16.915	0.268
Gap junction gamma-2 protein	GJC2	17.302	17.230	14.648	9.223
Gelsolin	GSN	11.059	11.298	11.620	2.481
Glucose-fructose oxidoreductase domain-containing protein 2	GFOD2	10.970	11.579	11.677	3.357
Glutamate-rich protein 6	ERICH6	10.997	11.266	10.520	3.456
HEAT repeat-containing protein 1	HEATR1	11.296	10.933	10.947	1.860
Heterogeneous nuclear ribonucleoprotein K	HNRNPK	11.416	11.408	10.766	3.328
Histone deacetylase 3	HDAC3	17.582	13.520	13.302	16.287
Histone H3.3	H3F3B	14.879	14.431	14.857	1.714
Histone H4	HIST1H4A	14.629	13.968	14.691	2.779
Histone-lysine N-methyltransferase SETD5	SETD5	12.961	13.833	12.021	7.004
Homeobox protein OTX2	OTX2	11.738	11.841	12.601	3.911
Homeobox protein PKNOX2	PKNOX2	10.556	10.893	10.424	2.277
Integrin alpha-D	ITGAD	15.100	19.642	19.639	14.462
Integrin beta	ITGB6	11.936	13.395	13.108	6.034
Interferon-related developmental regulator 2	IFRD2	11.446	11.267	10.441	4.849
Kelch-like protein 2	KLHL2	10.807	10.620	11.177	2.609
Latent-transforming growth factor beta-binding protein 3	LTBP3	15.041	11.669	13.195	12.692
Latent-transforming growth factor beta-binding protein 4	LTBP4	10.217	10.979	10.521	3.628
LIM/homeobox protein Lhx3	LHX3	17.029	17.059	17.678	2.122
Lipoma-preferred partner	LPP	14.404	13.912	13.789	2.321
MAP7 domain-containing protein 1	MAP7D1	12.861	12.890	12.624	1.139
Mediator of RNA polymerase II transcription subunit 31	MED31	13.143	12.187	13.291	4.653
Megakaryocyte-associated tyrosine-protein kinase	MATK	10.860	12.050	10.773	6.355
Melanoma antigen preferentially expressed in tumors	PRAME	10.544	11.745	9.985	8.361
Methyl-CpG-binding domain protein 1	MBD1	11.757	12.498	12.098	3.058
Methyl-CpG-binding domain protein 4	MBD4	11.041	10.915	10.845	0.908
Microfibril-associated glycoprotein 4	MFAP4	15.904	16.367	15.927	1.623
Microspherule protein 1	MCRS1	10.704	11.905	11.693	5.604
Mitogen-activated protein kinase kinase kinase 11	MAP3K11	12.451	12.374	11.430	4.707
Myosin light chain kinase, smooth muscle	MYLK	12.326	11.722	11.882	2.615

Myosin-10	MYH10	12.197	12.095	12.300	0.840
Myosin-11	MYH11	15.240	15.299	15.169	0.430
Myosin-9	MYH9	13.756	13.683	12.377	5.848
N-acyl-aromatic-L-amino acid amidohydrolase (carboxylate-forming)	ACY3	11.215	11.361	10.215	5.708
Nck-associated protein 5-like	NCKAP5L	19.236	16.247	19.186	9.392
Neuronal migration protein doublecortin	DCX	13.290	11.548	9.949	14.412
Neurotrophin-3	NTF3	11.492	11.191	12.188	4.399
NFAT activation molecule 1	NFAM1	11.396	10.861	10.703	3.303
Nicotinamide riboside kinase 1	NMRK1	11.748	10.678	11.771	5.479
NLR family member X1	NLRX1	11.145	10.877	11.486	2.734
NUAK family SNF1-like kinase 1	NUAK1	20.712	20.960	20.907	0.627
Nucleoprotein TPR	TPR	11.247	10.610	10.670	3.246
Nucleosome-remodeling factor subunit BPTF	BPTF	14.016	11.294	13.079	10.805
Olfactory receptor 2G3	OR2G3	14.423	13.081	14.804	6.417
Olfactory receptor 52B6	OR52B6	15.879	15.607	15.693	0.884
Olfactory receptor 6C3	OR6C3	18.198	17.111	18.572	4.224
Olfactory receptor 8S1	OR8S1	11.732	11.396	10.340	6.509
Palmitoyltransferase ZDHHC1	ZDHHC1	11.047	11.301	11.781	3.275
Pannexin-3	PANX3	10.796	10.475	10.712	1.560
PDZ and LIM domain protein 7	PDLIM7	12.951	12.640	11.761	4.955
Pecanex-like protein 2	PCNXL2	10.533	10.771	15.693	23.623
PHD finger protein 12	PHF12	12.437	10.227	11.178	9.832
Piezo-type mechanosensitive ion channel component 2	PIEZO2	17.001	17.091	17.102	0.324
Polypeptide N-acetylgalactosaminyl-transferase 2	GALNT2	9.708	9.741	10.163	2.569
PR domain-containing protein 11	PRDM11	10.597	11.282	11.202	3.396
pre-mRNA 3 end processing protein WDR33	WDR33	16.250	12.763	14.347	12.078
Probable E3 ubiquitin-protein ligase HERC4	HERC4	11.019	10.215	11.062	4.432
Probable ribosome biogenesis protein RLP24	RSL24D1	11.247	11.252	10.815	2.261
Proline and serine-rich protein 2	PROSER2	11.576	11.336	10.262	6.327
Proline-rich basic protein 1	PROB1	10.901	15.716	16.966	22.044
Proline-rich protein 11	PRR11	10.929	10.344	10.251	3.495
Protein FAM222B	FAM222B	11.407	10.962	12.213	5.501
Protein FAM3D	FAM3D	9.929	9.985	11.270	7.297
Protein jagged-1	JAG1	16.736	17.039	17.013	0.992
Protein maelstrom homolog	MAEL	21.203	21.278	20.777	1.282
Protein Tob2	TOB2	11.754	12.114	10.358	8.132
Protein transport protein Sec31A	SEC31A	17.072	17.002	16.982	0.277
Protein-tyrosine-phosphatase	PTPRQ	11.774	10.491	10.858	5.984
Putative cleavage and polyadenylation specificity	CPSF4L	14.772	16.924	13.743	10.716

factor subunit 4-like protein					
Pyrin	MEFV	17.510	17.580	17.770	0.762
Rab-like protein 6	RABL6	13.328	13.426	12.674	3.110
Ras association domain-containing protein 10	RASSF10	10.748	10.928	11.035	1.329
Ras-related protein Rab-7b	RAB7B	11.638	11.112	11.011	2.992
Ras-related protein Rap-1b	RAP1B	11.545	11.254	10.797	3.367
Receptor-type tyrosine-protein phosphatase O	PTPRO	11.322	11.383	11.831	2.416
Regulator of G-protein signaling 20	RGS20	19.680	19.654	19.686	0.086
Regulator of G-protein signaling 21	RGS21	13.270	13.885	12.306	6.052
Rho family-interacting cell polarization regulator 2	FAM65B	11.100	10.213	11.342	5.459
Rhombotin-2	LMO2	9.903	13.076	10.665	14.773
Serine/threonine-protein kinase 16	STK16	11.394	15.631	11.036	20.143
Serine/threonine-protein kinase 32B	STK32B	11.128	11.026	11.068	0.463
Serine/threonine-protein kinase ATR	ATR	10.806	11.409	10.833	3.091
Serine/threonine-protein kinase Nek8	NEK8	9.739	11.940	11.186	10.211
Serine/threonine-protein kinase TAO3	TAOK3	16.557	16.692	13.595	11.207
Serpin B13	SERPINB13	11.327	11.454	11.242	0.940
Sideroflexin-2	SFXN2	10.992	10.890	11.041	0.699
SLIT and NTRK-like protein 6	SLITRK6	18.185	18.401	18.498	0.874
Sodium/potassium/calcium exchanger 4	SLC24A4	10.459	10.211	11.721	7.498
Sodium/potassium-transporting ATPase subunit beta-2	ATP1B2	10.638	10.431	10.657	1.188
Sodium-dependent phosphate transporter 2	SLC20A2	16.207	21.061	20.722	14.018
Solute carrier organic anion transporter family member 5A1	SLCO5A1	13.182	15.511	10.866	17.612
Spermatogenesis-associated protein 13	SPATA13	11.353	11.610	12.105	3.269
Spermatogenesis-associated protein 24	SPATA24	10.518	11.242	10.802	3.359
Synaptotagmin-like protein 4	SYTL4	16.076	16.072	15.584	1.779
TBC1 domain family member 3B	TBC1D3B	13.750	13.949	14.074	1.174
Tetraspanin-31	TSPAN31	11.441	11.578	9.945	8.241
Tonsoku-like protein	TONSL	17.745	17.673	17.703	0.203
Transcription cofactor HES-6	HES6	10.863	10.919	9.919	5.320
Transcription factor COE1	EBF1	20.945	21.059	21.045	0.295
Transcription factor HIVEP3	HIVEP3	11.266	12.080	11.260	4.090
Transcription termination factor 1, mitochondrial	MTERF1	10.395	12.071	10.857	7.792
Transgelin	TAGLN	13.309	13.555	13.604	1.174
Transmembrane protein 87A	TMEM87A	19.829	19.888	19.906	0.202
TRIO and F-actin-binding protein	TRIOBP	15.877	10.529	15.643	21.565
Tropomyosin alpha-1 chain	TPM1	13.090	13.206	13.203	0.502

Tubulin alpha-1A chain	TUBA1A	13.480	12.121	13.282	5.664
Tubulin alpha-1B chain	TUBA1B	14.933	15.346	15.292	1.478
Tubulin beta chain	TUBB	14.153	14.182	14.059	0.455
Tubulin beta-2B chain	TUBB2B	10.559	12.245	10.851	8.034
Tubulin beta-4A chain	TUBB4A	10.728	11.217	11.897	5.204
Tubulin beta-4B chain	TUBB4B	11.907	12.053	11.736	1.334
Tyrosine-protein phosphatase non-receptor type 13	PTPN13	10.360	10.438	10.348	0.474
Vacuolar protein sorting-associated protein 54	VPS54	17.454	17.442	13.559	13.902
Versican core protein	VCAN	11.520	11.692	12.538	4.571
Vimentin	VIM	14.548	14.557	14.009	2.186
Voltage-dependent calcium channel subunit alpha-2/delta-3	CACNA2D3	11.129	10.930	10.824	1.414
Voltage-dependent L-type calcium channel subunit alpha-1C	CACNA1C	17.164	14.719	11.011	21.669
Voltage-dependent R-type calcium channel subunit alpha-1E	CACNA1E	11.264	10.916	11.148	1.598
WD repeat-containing protein 6	WDR6	15.313	15.271	15.379	0.354
WD repeat-containing protein 81	WDR81	10.997	11.650	11.030	3.277
Zinc finger and BTB domain-containing protein 17	ZBTB17	18.819	18.245	16.548	6.608
Zinc finger homeobox protein 2	ZFHX2	13.233	13.306	15.939	10.887
Zinc finger protein 493	ZNF493	11.531	12.064	11.770	2.266
Zinc finger protein 525	ZNF525	11.388	11.425	11.190	1.117
Zinc finger protein 778	ZNF778	11.088	10.774	12.039	5.827
Zinc finger protein with KRAB and SCAN domains 7	ZKSCAN7	10.142	10.724	11.323	5.506

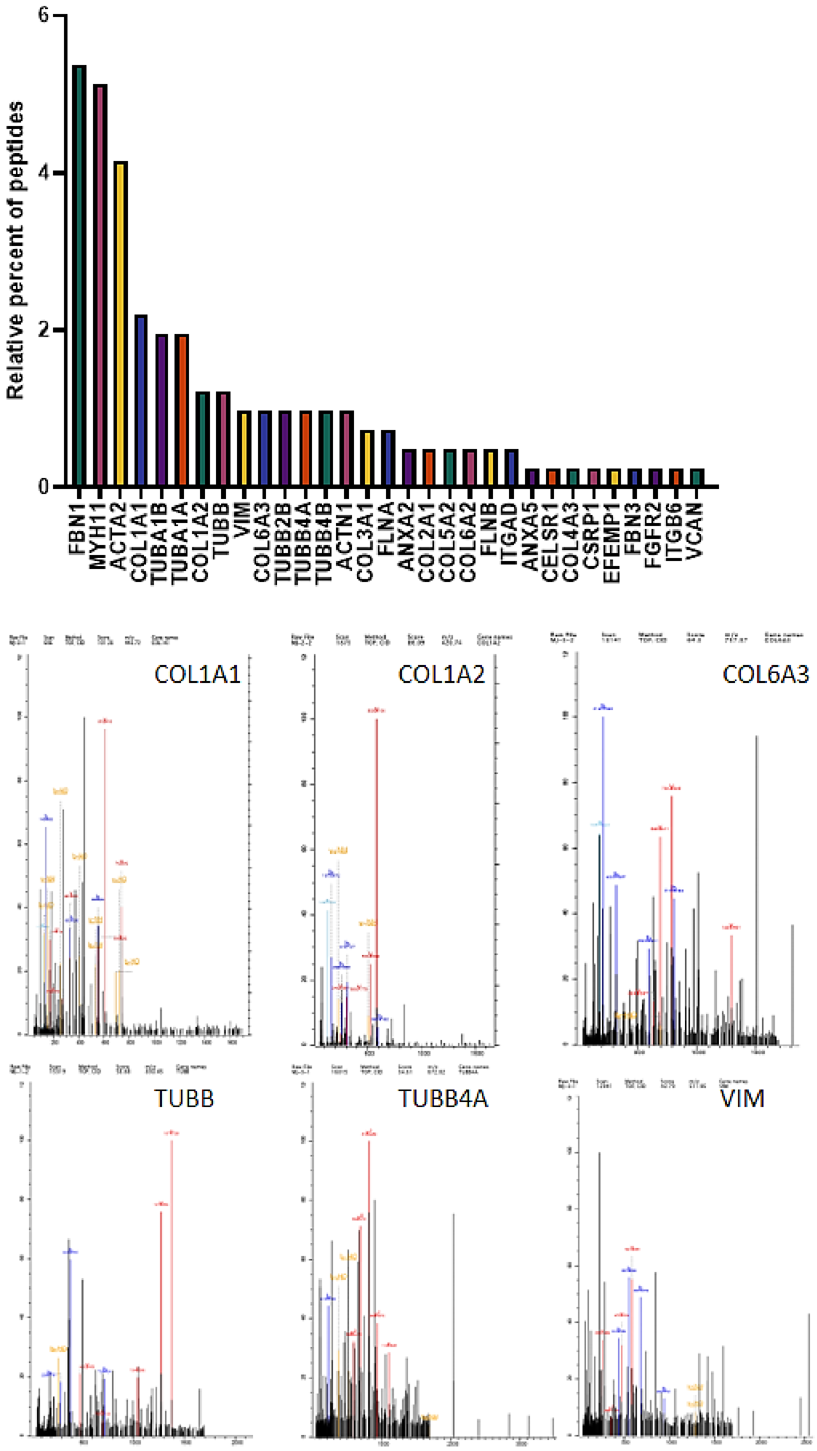

Figure S1. Bar graph representing the relative percentage of peptides and mass spectra related to some key ECM proteins as exemplars

2. Optimization of 3D-printability of three-component bioink

To optimize bioink for 3D-printing applications, we tested various component ratios of Alg, HAT, and dAECM. In addition, cross-linking time was seen as a critical parameter to construct robust and self-supporting hydrogels, that needed to be optimized. The optimized component ratio of Alg:HAT:dAECM was found to be 15%:4%:10 and cross-linking time were found to be 15%:4%:1% w/w/w, and the optimum cross-linking time was determined as 2h (**Figure S2**).

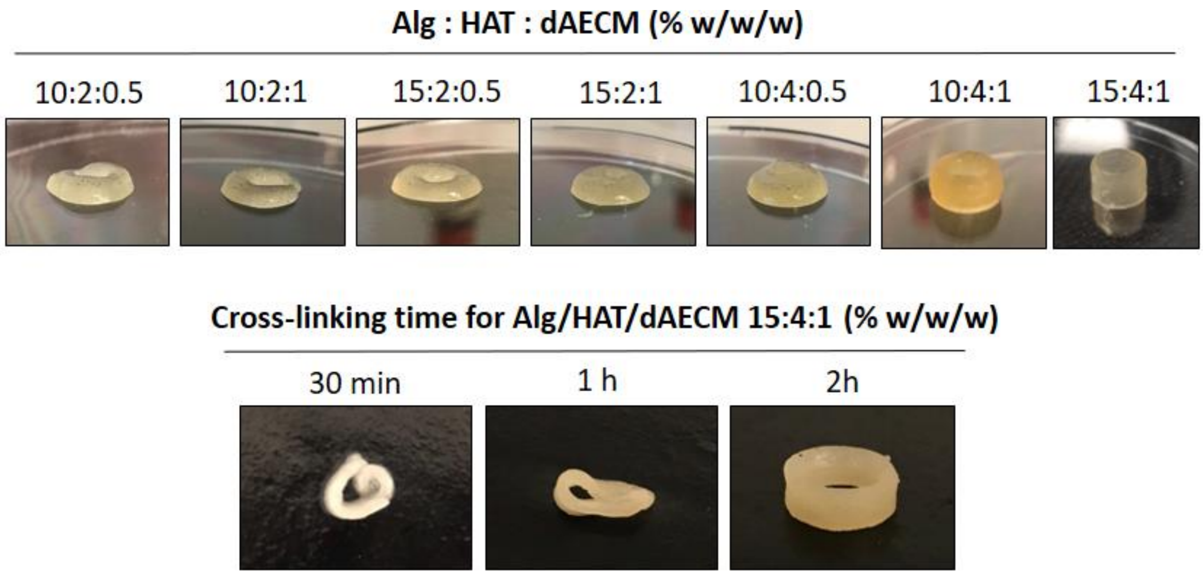

Figure S2. Different component ratio of Alg, HAT, and dAECM as well as cross-linking time for the optimized ratio of Alg:HAT:dAECM (15%:4%:1% w/w/w)

3. Spectroscopic assessment of three-component material and its components

To investigate the chemical structures of each component as well as the three-component composite material, we performed an FT-IR study. The spectrum relevant to the three-component structure exhibted unique bands combining individual components (**Figure S3**).

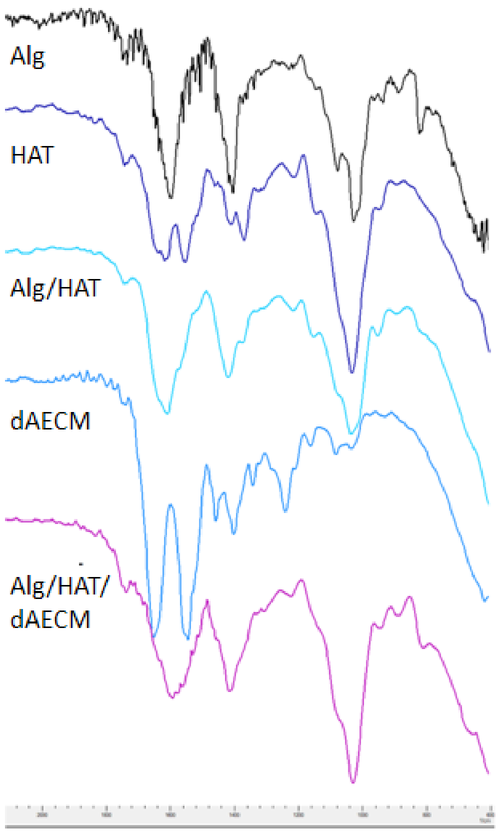

Figure S3. FT-IR spectra relevant to Alg, HAT, dAECM, Alg/HAT, and Alg/HAT/dAECM.

4. Assessment of viscoelasticity of hydrogels

Amplitude sweep tests were performed between 0.01 and 100% oscillation amplitude (shear strain) with a constant frequency of 1 Hz to determine the linear-viscoelastic range (**Figure S4**). Results showed that Alg is quite brittle, while combining HAT and dAECM enhanced the viscoelasticity of Alg-based constructs.

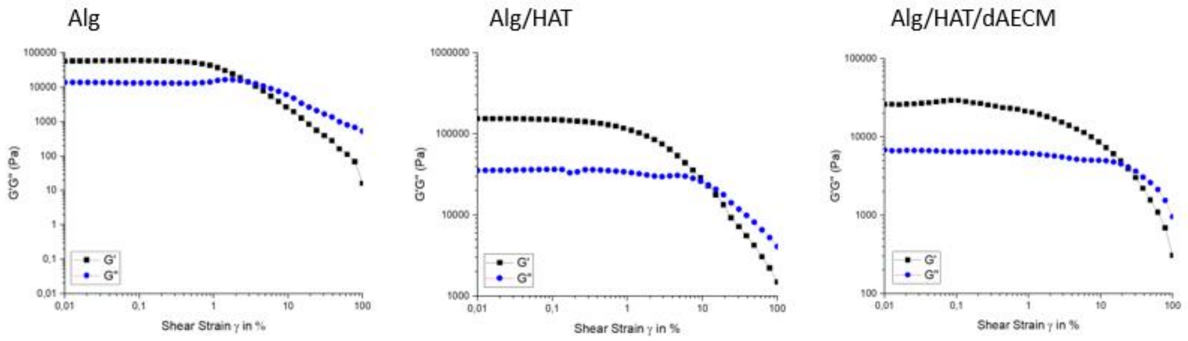

Figure S4. Amplitude sweep rheology for Alg, Alg/HAT, and Alg/HAT/dAECM

5. Structural fidelity of two-component constructs

To prove the indispensable role of each component, we prepared bioink formulations combining two components. To this aim, we tested the printability of Alg/HAT, Alg/dAECM, and HAT/dAECM (**Figure S5**). Alg/HAT formulation enabled a printing a large scale grid, however, it failed in the printing of vascular structure. On the other hand, Alg/dAECM emerged a structure with clear size and geometry, however, the resulting materials was brittle. As expected, when Alg was excluded from the formulation, HAT/dAECM formulation did not form a self-supporting construct, instead, it led to a hydrogel structure.

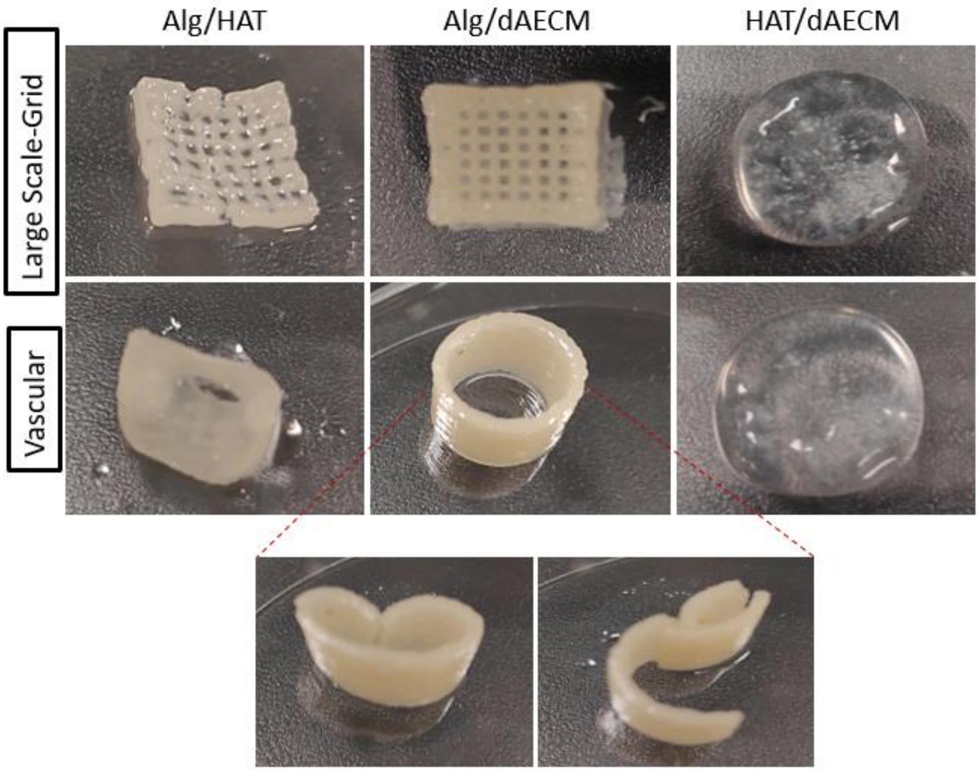

Figure S5. Structural fidelity of 3D-printed Alg/HAT, Alg/dAECM, and HAT/dAECM constructs.

6. The key role of Alg in the formulation

To investigate structural stability of the constructs and understand the key role of Alg in robustness, we immersed constructs in an aqueous solution of EDTA. The constructs were seen to turn into translucent and lost their shape fidelity (**Figure S6**). In contrast, multicomponent vascular construct immersed in PBS solution retained their texture, shape fidelity and dimensionality within the same timeframe.

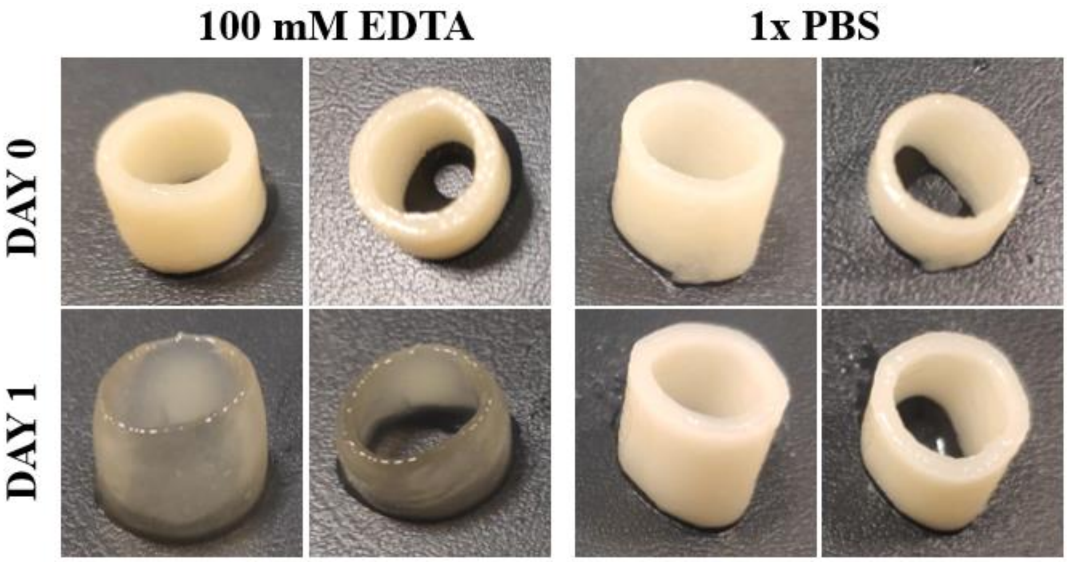

Figure S6. Structural stability of 3D printed multicomponent vascular constructs in EDTA solution and PBS.

7. Swelling test

The swelling analysis was conducted by immersing the freeze-dried and weighted (m_{dry}) scaffolds in PBS for 3 h at 37°C, and repeating the gravimetric measurements (m_{wet}). The swelling performance of the samples was determined by calculating the changes in mass according to the Equation (1) (**Figure S7**).

Swelling [%] = $[(m_{wet} - m_{dry}) / (m_{dry})] \times 100$ (Equation 1)

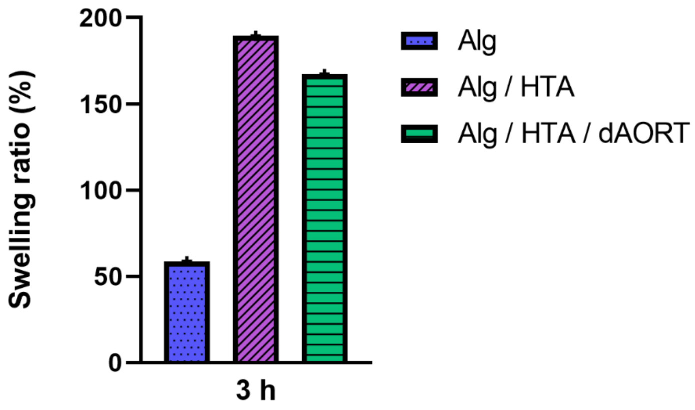

Figure S7. Swelling test

8. Cell proliferation test

To investigate the biological safety and cell proliferative capacity of Alg-based matrices, we performed an XTT testing for HUVECs seeded onto the materials. Results showed that Alg-based materials did not cause any toxicity and enabled a good proliferative ability (**Figure S8**).

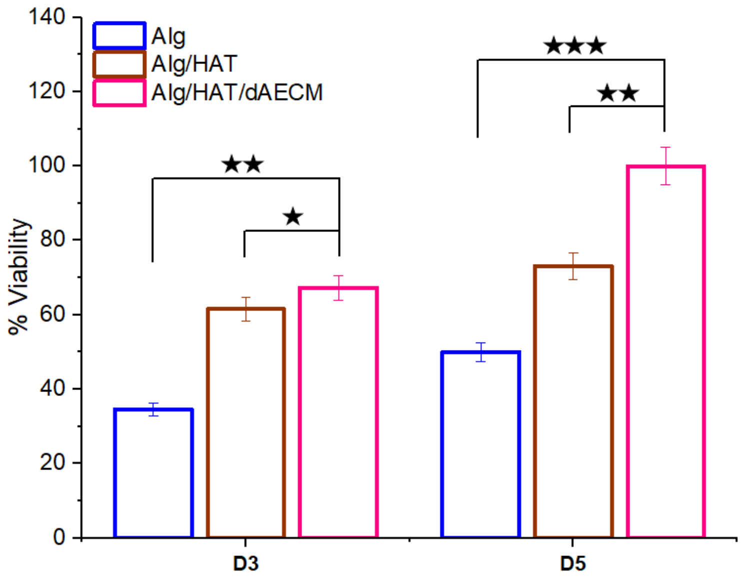

Figure S8. XTT test performed for HUVECs that were cultured atop **Alg**, **Alg/HAT**, and **Alg/HAT/dAECM** for up to 5 days (n=3, *p>0.05, **p<0.05, ***p<0.01).
